# A Quantitative LC–MS/MS Method for the Detection of 16 Synthetic Cathinones and 10 Metabolites and Its Application to Suspicious Clinical and Forensic Urine Samples

**DOI:** 10.3390/ph15050510

**Published:** 2022-04-22

**Authors:** Abdulaziz A. Aldubayyan, Erika Castrignanò, Simon Elliott, Vincenzo Abbate

**Affiliations:** 1 Department of Analytical, Environmental & Forensic Sciences, Faculty of Life Sciences & Medicine, King’s College London, London SE1 9NH, UK or aaldubayyan@pscc.med.sa (A.A.A.); erika.castrignano@kcl.ac.uk (E.C.); simon.elliott@kcl.ac.uk (S.E.); 2 Department of Toxicology, Central Military Laboratory and Blood Bank, Prince Sultan Military Medical City, Riyadh 12233, Saudi Arabia; 3 Elliott Forensic Consulting, Birmingham B16 9HN, UK

**Keywords:** synthetic cathinones, new psychoactive substances, NPS, LC–MS, urine

## Abstract

Background: Synthetic cathinones currently represent one of the most predominant (sub)-classes of new psychoactive substance (NPS) in illicit drug markets. Despite the increased concerns caused by the constant introduction of new analogues, these drugs are not commonly assayed in routine drug testing procedures and may not be detected in standard screening procedures. This study presents a validated liquid chromatography–tandem mass spectrometry (LC–MS/MS) method for the detection and quantification of 16 synthetic cathinones and 10 metabolites in human urine. Methods: The method was validated for all analytes using published guidelines. The evaluated parameters achieved acceptable values according to the set criteria. Potential abuse of synthetic cathinones was investigated in suspicious urine samples from Saudi Arabia originating from workplace drug testing, pre-employment and Accident & Emergency (A&E). Such samples generated a presumptive positive immunoassay for amphetamine; however, they yielded a negative LC–MS/MS confirmation for this analyte, following the recommended cutoff values of Substance of Abuse and Mental Health Services Administration (SAMHSA) guidelines. Results: 5.8% of the analyzed samples were found to contain at least one target analyte, namely mephedrone and *N*-ethylpentylone, as well as their dihydro-metabolites. The results also revealed polydrug use with the synthetic cathinones being present together with other classical stimulant drugs. Conclusions: This is the first report of NPS use in Saudi Arabia with respect to designer stimulant drugs. Confirmatory urine analyses for suspicious stimulant use should extend beyond classical stimulants to cover a broad range of NPSs and their metabolites in order to report any otherwise potentially undetected/new analyte.

## 1. Introduction

The rapid growth of clandestine laboratories over the last decade has resulted in a wide range of new psychoactive substances (NPS) [1], of which, a subgroup known as synthetic cathinones (SCts), or “bath salts”, has gained high popularity among consumers, possibly because of the expansion of online and virtual markets, cost and legal status [2,3]. SCts are synthetic derivatives of cathinone, which is one of the active ingredients found in the Khat plant (leaves of *Catha edulis*) [1]. Currently, the number of SCts reported for the first time to the European Monitoring Centre for Drugs and Drug Addiction (EMCDDA) over the past decade includes 156 analogues, which make up approximately 19% of the overall number of NPS [4]. Furthermore, the EMCDDA report in 2020 indicated that SCts are the most commonly detected substances among samples that are sold as NPSs [5]. In addition, the annual United Nations Office on Drugs and Crime (UNODC) report in 2020 showed that SCts were the most frequent NPS identified in the period of 2009–2019 [6].

As classical stimulants are routinely screened in many toxicological settings using immunoassay techniques, such as the enzyme-multiplied immunoassay technique (EMIT) or enzyme-linked immunoassay (ELISA), many SCts that share sufficient structural similarities may, thus, cross-react with the assay antibodies for classical stimulants. Several reports demonstrated the cross-reactivity of SCts in immunoassays targeting amphetamine, methamphetamine and/or MDMA [7,8,9]. Furthermore, a rise in the number of reports confirmed the presence of SCts, along with the latter classical stimulants, suggesting polydrug use associated with SCt consumption [10].

Current clinical and forensic laboratories are required to develop rapid and comprehensive methods to cope with the increasing number of newly introduced compounds to illicit drug markets. GC–MS was widely described in several studies for the investigation of SCts [11,12,13]. However, extensive sample preparation due to extraction, often followed by a derivatization step, is usually required [14]. Conversely, LC–MS techniques, using operational modes, such as MS/MS product ion scan or multiple reaction monitoring (MRM), may produce unique fragment ions from parent ions of the desired compound [15]. However, MS/MS may not easily discriminate between isomers, especially for compounds containing the same qualifier and quantifier mass transitions; in such cases, the LC component can aid in chromatographic separation, overall, achieving adequate specificity. In fact, the coupling of LC greatly improves detection accuracy when similar compounds are eluted at different times and if a certified reference material is available.

A large number of methods have been published using LC–MS/MS for quantitative analysis of SCt derivatives in biological samples such as urine [16,17,18,19,20], blood [19,20,21,22,23], oral fluid [24,25], fingerprints [26] and hair [27]. However, the emergence of new SCts in recent years may outdate previously reported targeted assays. Meanwhile, most of studies focused on developing targeted methods for the detection of parent compounds, with few or no metabolites being investigated.

**Figure 1:**
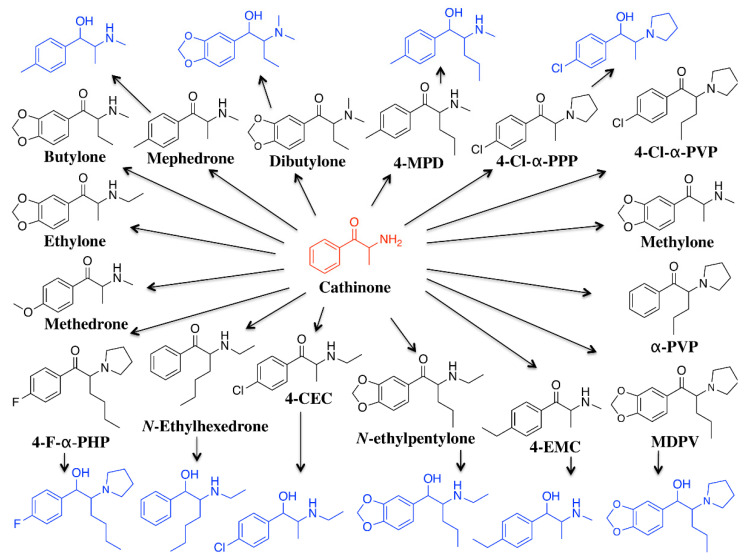
Chemical structure of synthetic cathinones (black) and reduced metabolites (blue) included in this study.

In this work, a quantitative method for the detection of a selection of relevant/emerging SCts and corresponding keto-reduced metabolites in urine using LC–MS/MS was developed and validated. The method was then employed to analyze a series of suspicious urine samples from Saudi Arabia that initially tested positive following immunoassay screening for amphetamine but for which the subsequent confirmatory test for amphetamine was negative for this analyte. We, thus, set out to determine the possible involvement of several SCts (Figure 1) in these samples. Moreover, the samples were further analyzed via LC–MS/MS to qualitatively screen conventional stimulants (methamphetamine, MDMA) to evaluate the use and prevalence of this class amongst classical drug users.

The selection of analytes to include in this study was difficult as there are no published data on the usage trends of specific SCts in Saudi Arabia. The selection of investigated SCts was, therefore, based on their forensic relevance and their prevalence in the globe from different generations since their emergence is still unclear in Saudi Arabia. For instance, mephedrone, dibutylone, α-pyrrolidinopentiophenone (α-PVP), methylenedioxypyrovalerone (MDPV), 4-chloroethcathinone (4-CEC), butylone, 4-chloro-α-pyrrolidinovalerophenone (4-Cl-α-PVP), 4-fluoro-α-pyrrolidinohexanophenone (4-F-PHP), 4-methylpentedrone (4-MPD), *N*-ethylpentylone and *N*-ethylhexedrone were among the SCt-related fatalities from 2017 to 2020, as reviewed by La Maida et al. [28]. Moreover, *N*-ethylhexedrone and dibutylone, two of the tested SCts, were also identified in Kuwait (a country that shares a land border with Saudi Arabia) [29]. In addition, since currently there are no published data related to the detection, identification and quantification of dihydro-4-chloro-α-pyrrolidinopropiophenone (dihydro-4-Cl-α-PPP), dihydro-*N*-ethylhexedrone, dihydro-4-ethylmethcathinone (dihydro-4-EMC) and dihydro-4-fluoro-α-pyrrolidinohexanophenone (dihydro-4-F-PHP) metabolites in human urine samples, these metabolites were included in this study.

## 2. Results

### 2.1. LC–MS/MS

**Figure 2:**
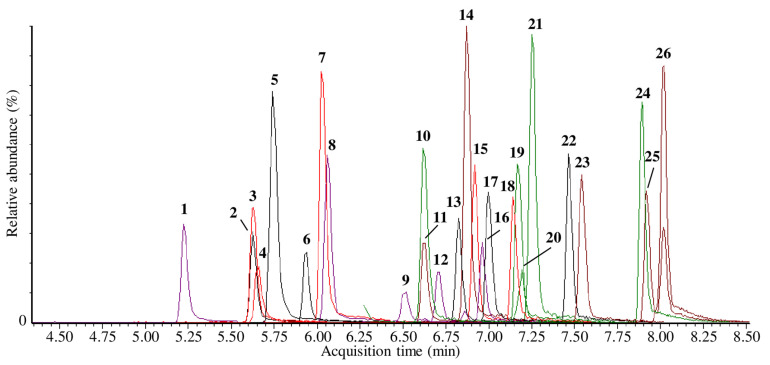
An overlay of extracted MRM chromatograms for all analytes. Analyte numbers are shown in Table 1.

An LC–MS/MS method for the detection and quantification of forensically relevant SCts and related metabolites in urine was developed and fully validated. Analytes were eluted within 8.2 min with a total run-time of 13 min. An overlay of extracted MRM chromatograms for all analytes is given in Figure 2, and corresponding analyte numbers are shown in Table 1.

### 2.2. Validation

#### 2.2.1. Linearity

Following initial, statistical evaluation of calibration models, the data were fitted to a linear regression model using the least square method with 1/x weighting factor. The method produced R^2^ > 0.99 for all analytes (Table 2).

#### 2.2.2. LOD and LOQ

**Figure 3:**
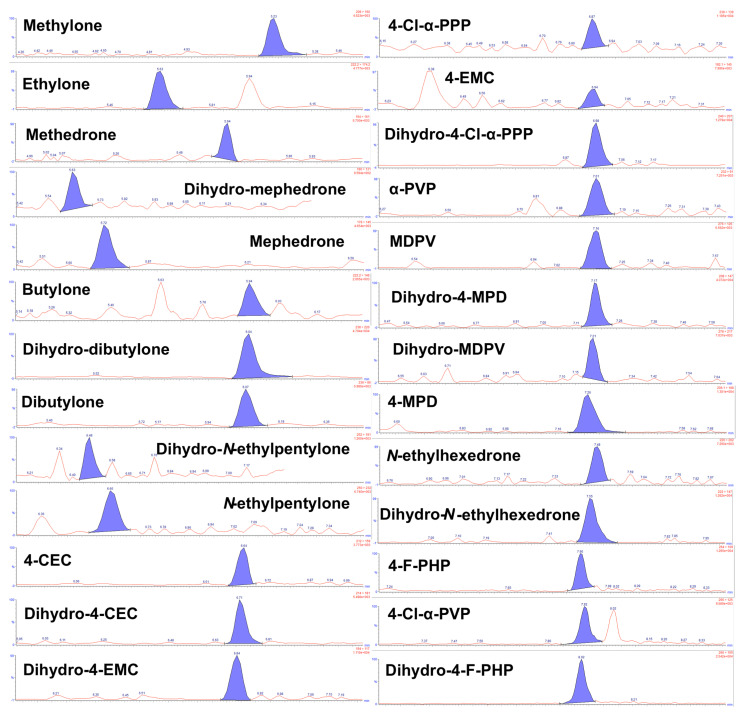
LC–MS/MS chromatograms of quantifier MRM transition (see Table 1) monitored for SCt analytes (*n* = 26) at the LOQ (1 ng/mL).

Limit of detection (LOD) ranged from 0.09 (4-CEC) to 0.5 ng/mL (dihydro-4-Cl-α-PPP). MRM chromatograms of the quantifier ions for all analytes in urine at the limit of quantification (LOQ) are shown in Figure 3. Calibration parameters, LOD and LOQ for all analytes are included in Table 2.

#### 2.2.3. Carryover

No peak was observed in the signal windows of target analytes in blank urine after the analysis of the highest calibrator (1000 ng/mL), which indicates that the method was free from carryover.

#### 2.2.4. Bias and Precision

Bias and precision at the three QC levels were within the acceptance values. Bias ranged from −16.9 to 9.5% of the true concentration. Within-run %CVs were: <12.9% (QC-low); <10.2% (QC-medium); <7.8% (QC-high). Between-run %CVs for the same concentrations were: <13.4%; <10.4%; <7.9%. Bias and precision at the three QC concentrations are summarized in Table 3.

#### 2.2.5. Interference

Interference from matrices, internal standard (IS) and other drugs was assessed. Blank urine (*n* = 12) samples collected from drug-free sources did not show interference with target analytes. The QC-high concentrations analyzed in the absence of IS did not reveal any peak associated with IS. No interfering peak associated with the signal of target analytes for blank matrices fortified with IS were observed. Blank urine samples fortified with commonly encountered analytes in clinical or forensic cases (Supplementary Table S1) were tested with the method. All analyzed samples did not show any peaks of the target analytes in their respective MRM channels.

#### 2.2.6. Matrix Effects

ME was found to be from 81.5 to 111.8% and 77.9 to 110.4% at 30 ng/mL and 800 ng/mL, respectively. The %CVs were <14.3% and <11.6% at 30 ng/mL and 800 ng/mL, respectively (Supplementary Figure S1).

Most of the analytes showed relatively low effects of ion suppression and enhancement (75.2 to 118.7%). However, the %CV of ME for all analytes was <15%, which suggested no critical variations of ME between samples.

#### 2.2.7. Processed Sample Stability

The stability experiments showed that the processed samples in urine were stable for 24 h, 48 h and 72 h when left on the autosampler at 10 °C. Table 4 presents the summarized results of processed sample stability.

### 2.3. Analysis of Authentic Urine Samples

**Figure 4:**
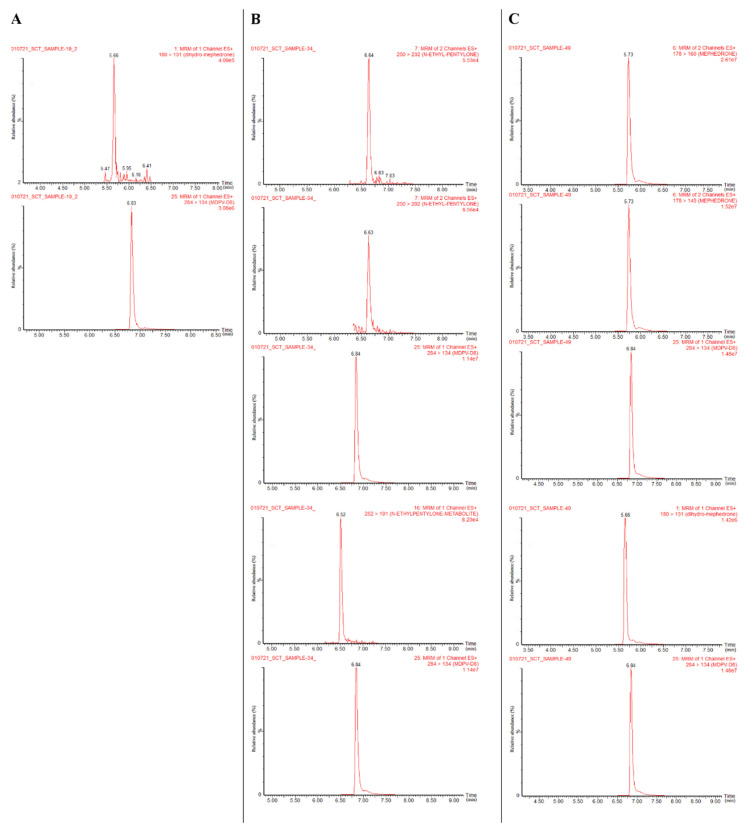
Results from the three authentic urine samples testing positive for target SCts: dihydro-mephedrone (sample no. 19) (**A**); *N*-ethylpentylone and dihydro-*N*-ethylpentylone (sample no. 34) (**B**); and mephedrone and dihydro-mephedrone (sample no. 49) (**C**).

A total of 52 real-case urine samples were successfully analyzed by the validated method. Of the samples tested, 5.8% were found to be positive for at least one target SCt included in this method, as illustrated in Table 5. Chromatograms of the three detected urine samples are shown in Figure 4.

The presence of metabolites was confirmed in accordance with Identity confirmation criteria, which provided unambiguous confirmation, as illustrated in Supplementary Figures S2–S4 and in Supplementary Table S2. Notably, dilution integrity was not performed as a part of the method validation. A concentration greater than the upper limit of quantification was observed for dihydro-*N*-ethylpentylone in sample no. 34 and mephedrone and dihydro-mephedrone in sample no. 49.

A dilution control was tested at 1000 ng/mL with a 1:5 dilution in blank urine samples. Bias results were within ±20% of the expected concentration for dihydro-*N*-ethylpentylone (–3.4%) and mephedrone (17.1%) except for dihydro-mephedrone (–26.6%). Consequently, dihydro-*N*-ethylpentylone and mephedrone were diluted and re-analyzed; however, dihydro-mephedrone was reported as ‘above the upper limit of quantification’ even after dilution.

## 3. Discussion

A thorough sample clean-up procedure is a crucial step to take before utilizing hyphenated techniques to minimize matrix effects and aid selectivity. Initial experiments showed that an LLE method could be applied for SCt detection. This extraction method was also applied to extract a broad range of chemically basic analytes that had varying physiochemical properties [30].

LC–MS/MS enables the detection of target analytes with increased sensitivity and selectivity. This is particularly important for SCts and other related NPS which may be present at lower biological fluid concentrations than other conventional drugs (e.g., amphetamine). Unique MRM transitions were carefully reviewed to ascertain all product ions were explainable as proposed fragments of the target analytes. Coupled with LC separation, this provided a robust analytical system for unambiguous analyte detection. However, it was found in preliminary experiments that butylone and ethylone were the most difficult analytes to separate chromatographically. Moreover, both analytes generate similar MS/MS MRM transitions that do not allow conclusive identification and quantification. O’Byrne et al. [31] reported these analytes as ethylone/butylone owing to the difficulties in separating them as different analytes. This analytical issue encountered is not surprising because, with the increased number of SCts, structural isomers and isobarics increase the number of analytes that exhibit common transitions, which may challenge both the separation and detection of target analytes [32]. However, Ploumen et al. [33] successfully separated SCt isomers by lowering the column temperature. In this work, employing the chemistry of the HSS T3 column, along with lower temperature (from 30 °C to 20 °C), provided the best separation for both analytes. Despite identical MRM transitions, baseline separation was enough to permit correct quantification. Ethylone and butylone eluted at 5.62 min and 5.94 min, respectively (Supplementary Figure S5). Although MRM methods allow simultaneous quantification of several analytes in one analytical run, the number of measured analytes is not unconstrained but tied to the following aspects: (1) an optimal run time; (2) the number of monitored MRM channels per function; (3) the total number of monitored transition ions in the method; (4) a considerable dwell time, having a large influence on the acquired data points across the chromatographic peak. Notably, the MS analyzer used in this work did not allow the introduction of a large number of transition ions into the method and, consequently, resulted in a low number of data points across the peak, especially in regions of co-elution of several analytes. It was, thus, necessary to monitor only one MRM transition for the employed metabolites; nevertheless, a second transition is normally desirable to confirm the identity of an analyte. Therefore, for confirmation purposes, a sub-method was created, containing two MRM transitions for each metabolite. This sub-method had an identical LC–MS/MS condition to the primary method and should only be used in the presence of metabolites for confirmatory purposes. The dwell times were optimized to ensure 12–20 data points per chromatographic peak, achieving adequate quantification capabilities for each analyte.

Detection of metabolites in matrices may further demonstrate drug intake and, in some instances, metabolites/drug ratios may provide the approximate time of drug consumption or changes in metabolism [17]. Several studies reported the detection of SCts in various matrices [13,19,23,34]. However, the majority of these studies only included a few (no more than six) or no metabolites. Despite no previous data being available for the detection of other metabolites, the reduction of the ketone group to form alcohol moieties is likely expected as one of primary routes of biotransformation [35]. The presented method included 10 selected, reduced SCt metabolites, including dihydro-4-Cl-α-PPP, dihydro-*N*-ethylhexedrone, dihydro-4-EMC and dihydro-4-F-PHP, not previously established in toxicological urine analyses.

It is noteworthy that the reduced metabolites were detected in all positive samples (in addition to the parent). Dihydro-mephedrone metabolite, however, was solely detected in sample no. 19. The addition of mephedrone and *N*-ethylpentylone-reduced metabolites supported the method’s capability to analytically confirm the parent use, consolidating an extended window of detection when targeting urinary metabolites. In related work, dihydro-mephedrone was detected in human urine and blood samples [36,37].

Fan et al. [34] determined the presence of mephedrone and its metabolite (dihydro-mephedrone) in 11 out of 18 samples in urine from forensic cases, of which metabolites were only detected in four samples. Furthermore, testing metabolites can aid the extension of the detection window, especially when the instability of parent is a concern. This is particularly true in the case of SCts as, sometimes, only metabolites are detected in biological samples. In the stability investigation of 4-methylethcathinone (4-MEC), Soh and Elliott [30] found that dihydro-4-MEC, resulting from keto reduction to an alcohol, was the prominent metabolite found in a forensic casework sample and, in the absence of 4-MEC itself (e.g., following in vitro and ex vivo sample instability), could serve as an appropriate marker to determine the intake of 4-MEC. In our work, the measured levels of dihydro-metabolites were considerably higher than their parent, which may explain the presence of dihydro-metabolites in the absence of their parent in previously published methods. For these reasons, the presence of reduced metabolites may occur in biological samples, and it is recommended, therefore, to monitor these analytes in the tested panel for the confirmation of the intake of the parent drug.

The remaining 94.2% (49 of 52) of the analyzed samples was negative for SCts, suggesting no consumption or use of SCt analogues not included in this study. Indeed, it is possible that other SCts were consumed and not detected with this method given the fact that newer types of SCt constantly emerge into the market. It is also likely that target analytes were present in the samples at very low levels (<LOD) owing, for instance, to their degradation in the matrix. Indeed, samples were stored from one to six months, and there is evidence strongly suggesting the tendency of this class to degrade over time, even when stored at low temperatures [38].

SCts are often identified in combination with traditional stimulants, which may lead to neurotoxicity or other unexpected toxicity profile. For instance, evidence shows that mephedrone does not damage dopamine striatal nerve endings by itself, but it does boost the neurotoxicity of amphetamine, methamphetamine and MDMA [39]. Hence, it was decided to further screen all samples via LC–MS/MS to determine the presence of traditional stimulants, such as methamphetamine and/or MDMA, despite it not being part of the scope of the developed method. Although no validation was performed, two product ions were deemed sufficient to allow target analyte identification. MRM transitions of methamphetamine and MDMA were chosen according to the literature [40]: transitions for methamphetamine *m*/*z* 150 → 119, 150 → 91 and, for MDMA, *m*/*z* 194 → 163, 194 → 105 using MDPV-*d*_8_ as IS.

Methamphetamine was detected in all positive samples (no. 19, 34 and 49), while MDMA, in addition to methamphetamine, was detected in sample no. 49. The presence of methamphetamine and/or MDMA is in agreement with the finding that, in many occasions, SCts are frequently consumed in combination with other drugs of abuse [31]. Moreover, it is also possible that SCts may be consumed unintentionally as a result of adulterants or replacements for substances sold as classical stimulants [41]. Whether these substances were mixed with classical drugs or whether different substances were simply ingested, polydrug intake can increase the risk of toxicity, overdose and/or fatality [42].

Amphetamines are some of the most commonly encountered drugs in toxicological cases involving drugs of abuse. Therefore, they are commonly included in urine immunoassay screening of suspected drug abuse [43]. EMIT II Plus amphetamine immunoassay is intended for the detection of amphetamine and methamphetamine in human urine [44]. However, amphetamine and methamphetamine are structurally rather simple, which makes it challenging to develop specific antibodies targeting these drugs. In addition, there are many chemically related drugs, which may increase the likelihood of false positive results in an initial drug screen [45]. As SCts are structurally related to amphetamines, it was hypothesized that some target analytes may cross-react with amphetamines in immunoassay screening. However, other amphetamine derivatives, especially methamphetamine, were positive in all SCt-positive samples and, therefore, it is difficult to conclude the contribution of target SCts to the immunoassay cross-reaction. Signal intensities from the chromatograms of samples no. 34 and 49 demonstrated potential high concentrations of methamphetamine. Such results were interpreted as a typical cross-reaction for which the assay is essentially intended. It should be taken into account for the potential involvement of other interfering drugs not covered by the LC–MS method or screenings for classical stimulants, which could account solely or partially for cross-reactivity. This might be the case for sample no. 19, since dihydro-mephedrone was identified at a low concentration, as well as certain traces for methamphetamine and MDMA. In previously published work involving an ethylone fatality, a few samples revealed immunoassay cross-reaction with amphetamine and MDMA, but none was detected in confirmatory testing, and the authors also observed no cross-reaction in other samples despite them having higher ethylone concentrations [7]. Thirakul et al. [46] reported a ‘presumptive’ positive immunoassay for amphetamine/methamphetamine, but, subsequently, it was confirmed negative via GC–MS; further investigations determined the presence of *N*-ethylpentylone and other drugs.

This study had some limitations, for instance, the selection of only one IS (MDPV-*d*_8_) for all the analytes. In initial experiments, three deuterated IS were selected (MDPV-*d*_8_, mephedrone-*d*_3_ and α-PVP-*d*_8_) based on the functional group similarity of investigated analytes, although, ideally, a deuterated analogue for each analyte should have been added to appropriately compensate for ME. However, this was not possible due to the commercial availability and cost for the extensive list of SCts. Mephedrone-*d*_3_ and α-PVP-*d*_8_ were later excluded due to the falsely elevated peak area with increased analyte concentrations. This phenomenon was not observed for MDPV-*d*_8_ at 500 ng/mL for all analytes and, therefore, was the sole IS used in the method. Another limitation was the selection of investigated SCts, considering the evolving number of new drugs reported each year, and, thus, could potentially explain the low number of detected SCts in comparison to traditional stimulants. Nevertheless, an important outcome of this study was represented by the discovery of the presence of SCts in Saudi Arabia, which would otherwise not have been confirmed with the traditional approaches and confirmation methods that do not commonly include NPSs. This finding indicates that SCt use is a continuing, global problem, and Saudi Arabia is not an exception.

## 4. Materials and Methods

### 4.1. Chemicals and Reagents

Reference standards, including 4-ethylmethcathinone (4-EMC), MDPV, 4-CEC, α-PVP, methylone, *N*-ethylpentylone, methedrone, ethylone, *N*-ethylhexedrone, butylone, dibutylone and mephedrone, were obtained as hydrochloride salts from Chiron (Surrey, UK). Deuterated IS MDPV-*d*_8_ (hydrochloride) was obtained from Sigma-Aldrich (Dorset, UK). 4-MPD, 4-F-PHP, 4-chloro-α-pyrrolidinopropiophenone (4-Cl-α-PPP) and 4-Cl-α-PVP were kindly donated by TicTaC communications (London, UK) and test-purchased online and analyzed via high-resolution mass spectrometry (HRMS) for their identity. All reference and deuterated standards were obtained as methanolic solutions at 1.0 or 0.1 mg/mL, except for mephedrone, 4-MPD, 4-F-PHP, 4-Cl-α-PPP and 4-Cl-α-PVP (available as powder).

Ultra-pure water (18.2 MΩ cm) was generated in house using a Millipore water purification system. All solvents were HPLC grade unless otherwise stated. Methanol (MeOH) and acetonitrile (ACN) were obtained from Sigma-Aldrich (Dorset, UK). 1-Chlorobutane and sulfuric acid (≥95%) were both obtained from Thermo Fisher Scientific (Loughborough, UK).

### 4.2. Preparation of Standards and Solutions

Working solutions of calibration standards consisting of all 26 analytes and keto-reduced metabolites (dihydro-) were prepared in MeOH at 2, 10, 20, 40, 100, 1000, 1500 and 2000 ng/mL, whilst those used for the quality controls (QC) were prepared at 60, 800 and 1600 ng/mL. The IS solution containing MDPV-*d*_8_ was prepared in MeOH at 2 μg/mL.

### 4.3. Synthesis of Reduced Metabolites

SCts are metabolized to several phase I metabolites, one of which is their keto-reduced metabolite (dihydro-), which is also an instability product [30]. However, many such metabolites are not commercially available to be used as reference standards. Dihydro-metabolites were, therefore, synthesized from parent analytes (Supplementary Figure S6), in which the keto group was reduced to alcohol following a previously described method [47]. As a proof of this approach, the reduction of mephedrone to dihydro-mephedrone was studied as a starting analyte, and the results were compared with previously published data [36]. Briefly, the experimental synthetic procedure was as follows: 8 mg of sodium borohydride was added carefully and in small portions to a solution of mephedrone (0.4 mg, 2.25 × 10^−6^ mmol) in MeOH (4 mL). The solution was left overnight with agitation at room temperature. Then, the resultant mixture was dried under vacuum, and the solid residue was partitioned in dichloromethane/water (4 mL), and the organic layer was extracted (2 × 3 mL of water). Thereafter, 20 mg of sodium sulfate was added to the combined, isolated organic layer, the solution was filtered and dried under vacuum, leading to dihydro-mephedrone (0.000404 g, 2.25 × 10^−6^ mmol calculated as a theoretical 100% yield based on literature findings and due to inability to accurately measure the amount of product). The solid residue was dissolved in 4.04 mL MeOH to achieve an estimated 100 μg/mL stock solution. The product ion spectra were identified by HRMS and were in accordance with reported literature. Therefore, the method was applied to the following selected analytes: dibutylone, 4-CEC, 4-Cl-α-ppp, 4-EMC, *N*-ethylpentylone, MDPV, 4-MPD, *N*-ethylhexedrone and 4-F-PHP.

### 4.4. Liquid–Liquid Extraction (LLE)

Calibration standards at 1, 5, 10, 20, 50, 150, 500, 750 and 1000 ng/mL were prepared by adding an appropriate volume of the standard working solution to urine. Likewise, QC-low (30 ng/mL), QC-medium (400 ng/mL) and QC-high (800 ng/mL) were prepared by the addition of an appropriate volume of the QC working solution to urine. An appropriate volume of IS at 2 μg/mL was added in 0.2 M sodium carbonate solution to achieve final concentration of 500 ng/mL.

LLE was performed with the addition of 500 μL of sodium carbonate (pH = 10) containing IS to 500 μL urine containing standard/QC. To each sample, 5 mL of 1-chlorobutane was added in 15 mL polypropylene tubes. For the ‘zero’ standard sample, 500 μL of sodium carbonate containing IS was added to 500 μL urine in the absence of standard/QC. For blank samples, 500 μL of sodium carbonate aliquot was added to 500 μL of blank urine samples. All samples were vortexed thoroughly for 3 min and centrifuged for 5 min at 3000 rpm and then frozen for 30 min at −40 °C. After freezing, the top layer of all samples was decanted to new 15 mL polypropylene tubes. Samples were back-extracted by adding 100 μL of 0.05 M sulfuric acid to the top (organic) layer and then re-mixed for 3 min followed by centrifugation for 5 min at 3000 rpm. Samples were placed in the freezer for 30 min at –40 °C. After removal from the freezer, the top layer of samples was discarded, and the aqueous acid (bottom) layer was allowed to thaw for 5 min at room temperature. Finally, the acid layer was transferred into an HPLC vial ready for injection.

### 4.5. LC Instrument

Analysis of samples was performed on an Acquity UPLC^®^ system (Manchester, UK) equipped with a HSS T3 UPLC analytical column (150 × 2.1 mm, 1.8 μm) (Waters) maintained at a temperature of 20 °C, and the pump was operated at a flow rate of 0.30 mL/min. A binary gradient system was used to separate analytes consisting of mobile phase A, 0.1% (*v/v*) formic acid (FA) in ultra-pure water and mobile phase B, 0.1% (*v/v*) FA in ACN. The gradient profile started at 90% A (0–1.80 min), decreased to 64% A (1.80–6.0 min) and then further decreased to 0% A (6.0–9.80 min). This was maintained for 1 min (9.80–10.80 min). Within 0.1 min, A was returned to initial condition, i.e., 90% (10.80–10.81 min), and kept until the end of the run (10.81–13.00 min) to re-equilibrate the column.

The injection volume was 10 μL, using partial loop with needle overfill. The UPLC system was equipped with two needle wash solution reservoirs: weak needle wash (0.2% formic acid in water:ACN 70:30 *v/v*) and strong needle wash (MeOH:ACN 90:10 *v/v*), which were used to clean the needle and wash station before each injection to eliminate any possible remaining contaminants from the needle and to prevent any pre-column-related carryover effects. Water:ACN (90:10, *v/v*) was used as seal wash solvent.

### 4.6. MS Conditions

Target analytes were detected using a Waters Quattro Premier XE™ Triple Quadrupole (QqQ) Mass Spectrometer System (Manchester, UK) operating in positive electrospray ionization mode (ESI+). Data acquisition and analysis were performed using MassLynx v. 4.1 (Waters). The electrospray voltage was set at 1.5 kV (ESI+). The desolvation and source temperatures were set at 400 and 120 °C, respectively. Nitrogen was employed as the desolvation and cone gas, which were set at 750 L/h and 50 L/h, respectively. Argon was employed as the collision gas at a flow rate of 0.2 mL/min, which typically gives pressures of 2.14 × 10^−3^ mbar. MRM transitions were developed and optimized for each target analyte via combined post-column infusion mode. Further, MRM was used to monitor two transition ions for parent analytes (one as quantifier and one as qualifier) and one for reduced metabolites (as quantifier) and IS. As the first choice, the most abundant product ion was used for quantification, and, where applicable, the second most abundant one for qualification purposes. However, in some cases, when analytes produced similar product ions upon fragmentation, a second transition was selected.

The dwell time was optimized for each analyte to achieve at least 12 data points per peak. Retention time (Rt), precursor ions, product ions, cone voltages (CVs), collision energies (CEs) and dwell times for each analyte are detailed in Table 1. Data were acquired with MassLynx 4.1 software and processed with QuanLynx 4.1 software.

### 4.7. Confirmation of Positive Findings

Identity confirmation criteria for target analytes in real case samples at concentrations greater than the LOQ included the presence of two selected product ions; the relative retention time of the target analyte to the corresponding IS, which should comply with that of the spiked sample within a tolerance of ±2.5%; and the ion ratio between the less intense peak area and that of the more intense, which was established by the spiked QC samples, should comply within the permitted tolerance stipulated in Supplementary Table S3 [48].

### 4.8. Validation Procedure

The method was validated utilizing the recommendations and guidelines of the Scientific Working Group of Forensic Toxicology (SWGTOX) that evolved into ANSI/ASB standard 036 [49,50]. The evaluation of the quantitative method included the following parameters: linearity, bias, precision, carryover, LOD, LOQ, matrix effects, interference and stability. The validation parameters were calculated by introducing the corresponding formulae into Microsoft Excel.

#### 4.8.1. Linearity

The calibration curve was assessed by analyzing eight non-zero calibration points (1, 5, 10, 20, 50, 150, 500 and 1000 ng/mL for parent analytes; 1, 5, 10, 20, 50, 150, 750 and 1000 ng/mL for metabolites). Peak area ratios (PAR) of analytes and IS were calculated as follows (Equation (1)):

PAR = peak area of analyte/peak area of IS(1)

Values in coefficient of determination (R^2^) should be ≥0.990 to meet the acceptance criteria.

#### 4.8.2. Carryover

Blank matrix samples (i.e., extracted urine without any SCt addition) were analyzed (*n* = 3) after the highest calibrator (1000 ng/mL) to assess for any carryover. The analyses of blank with no target peak exceeding its LOD was deemed free from carryover.

#### 4.8.3. Bias and Precision

Bias and precision were assessed at three concentrations: QC-low (30 ng/mL), QC-medium (400 ng/mL) and QC-high (800 ng/mL) for five independent runs. Bias and precision were evaluated using replicates (*n* = 3) of spiked matrix, along with the batch of calibration curves. Bias was calculated using Equation (2):

Bias (%) = (grand mean of calculated concentration–true concentration/true concentration) × 100(2)

Within-run precision was carried out for each concentration (*n* = 3) of each run, as well as within- and between-run precision for all the runs (*n* = 15). Both precisions (also expressed as coefficient variation (%CV)) were determined from the data generated for calculating the bias using Equations (3) and (4):

Within-run precision (%CV) = (SD of single run (triplicate analyses)/mean of single run) × 100(3)

Between-run precision (%CV) = (SD of grand mean for each concentration/grand mean of calculated concentration) × 100(4)

The acceptable value was that bias and precisions should be ≤±20% at each concentration. One-way ANOVA was used to facilitate calculation of precision.

#### 4.8.4. LOD and LOQ

LOD was the lowest concentration at which analytes could produce a signal-to-background noise (S/N) ratio of 3:1 or more. LOQ was defined as the lowest concentration of analyte that could reliably produce quantitative results with a signal-to-noise (S/N) ratio of 10:1 or more. The lowest concentration of calibration curve (1 ng/mL) was assigned as the LOQ of the method.

#### 4.8.5. Interference

Interference from matrix components was evaluated using extracted, drug-free matrix in the absence of analytes and IS. Due to the potential application of this method on real samples, samples were collected from a minimum of 10 drug-free individuals. Interference generated from the use of IS was assessed using spiked, drug-free urine with IS (500 ng/mL) and by monitoring the MRM peaks of the target analytes. Similarly, interference associated with analytes was evaluated using the highest calibrator (1000 ng/mL) in the absence of IS. Drug interference was assessed using two groups of control solutions (kindly donated by the Drug Control Centre, King’s College London) consisting of a wide range of commonly encountered analytes (*n* = 196). Each control solution of A and B groups (10 or 100 ng/mL) was fortified with drug-free urine in the absence of SCt analytes and IS to ensure no false-positive signals of target analytes. It should be noted that control solution B also contained mephedrone; thus, this analyte was not considered for its selectivity. A full list of analytes included in the interference study are shown in Supplementary Table S1.

#### 4.8.6. Matrix Effects

Matrix effects (ME) were quantitatively assessed using a post-extraction method described in the SWGTOX guideline [49]. Two different sets of samples were prepared for urine at QC-low (30 ng/mL) and QC-high (800 ng/mL). Set A consisted of neat methanolic solution standards (*n* = 5); set B consisted of samples (*n* = 5) that were spiked with target analytes post extraction. Set B was prepared from the urine of five drug-free individuals. ME was determined by comparing the average peak area of analytes in set A and B (Equation (5)).

ME (%) = B/A × 100(5)

ME values of <100% demonstrate ion suppression, and values of >100% demonstrate ion enhancement. ME values of ≤25% demonstrate ion suppression or enhancement, with %CV ≤ 15% deemed to be acceptable.

#### 4.8.7. Processed Sample Stability

The stability of processed samples was assessed by extracting samples at QC-low and QC-high (*n* = 3). Samples were immediately analyzed to establish day-zero concentration and left cooled (10 °C) in the autosampler for re-analyses after 24, 48 and 72 h. The average peak area of analytes was compared with those of day zero and was reported as stable until results exceeded acceptable bias (>±20%).

### 4.9. Authentic Urine Samples

A total of 52 authentic urine samples were collected between September 2020 and April 2021 from workplace drug testing, pre-employment and A&E in collaboration with Prince Sultan Military Medical City (PSMMC) (Riyadh, Saudi Arabia). As per PSMMC’s routine protocols, samples were initially tested by immunoassay techniques for drugs of abuse, including amphetamine, cannabis and opiates. This study only employed urine samples that produced positive result to amphetamine using EMIT II Plus immunoassay at 500 ng/mL but, subsequently, confirmed negative to amphetamine via LC–MS/MS as they did not exceed the SAMHSA threshold. No information regarding the source of samples was provided since samples were anonymized prior to shipment as per approval given by the Ethics Committee. Storage and shipment conditions of urine samples are summarized in Table S4.

## 5. Conclusions

With the wide spectrum of available designer stimulants, including SCts, toxicology laboratories must be able to accurately detect and quantify these analytes. In addition, the ability of existing methods to determine SCts, including their metabolites, has been poorly studied. In this work, a sensitive and selective LC–MS/MS method was developed and validated to quantitatively identify 16 SCts and 10 metabolites in urine samples. Good sensitivity was provided with relatively low sample volume of 500 μL and limits of detection ranging from 0.09 to 0.5 ng/mL. Under the chromatographic conditions described in this work, separation of isobaric analytes (ethylone and butylone) allowed a correct quantification if both analytes were present in the same sample. Incorporation of metabolite data could help in the interpretation of results of these analytes in both forensic and clinical laboratories. The application of this method demonstrated polydrug consumption of traditional and new, synthetic stimulant drugs in Saudi Arabia, which was not reported before and supports the importance of updating confirmatory testing to include NPS and new stimulant drugs in particular.

## Figures and Tables

**Table 1:** Retention time (Rt), precursor ion, product ions (quantifier ions in bold), cone voltage (CV), collision energy (CE) and dwell time for each target analyte.

Analyte No.	Target Analyte	Rt (min)	Precursor Ion (m/z)	Product Ion(s) (m/z)	CV (V)	CE(eV)	Ion Ratio	Dwell Time (s)
1	Methylone	5.23	208	**160**	22	17	0.27	0.150
				132		25		
2	Ethylone	5.63	222	**174**	24	19	0.18	0.050
				146		24		
3	Methedrone	5.63	194	**161**	23	19	0.34	0.010
				176		12		
4	Dihydro-mephedrone	5.64	180	**131**	24	18	-	0.050
				147 *		19		
5	Mephedrone	5.72	178	**145**	24	20	0.52	0.050
				160		12		
6	Butylone	5.94	222	**146**	24	24	0.28	0.020
				204		13		
7	Dihydro-dibutylone	6.02	238	**220**	26	12	-	0.050
				191 *		20		
8	Dibutylone	6.07	236	**86**	26	21	0.60	0.050
				191		15		
9	Dihydro-*N*-ethylpentylone	6.49	252	**191**	27	23	-	0.010
				234 *		15		
10	*N*-ethylpentylone	6.60	250	**202**	27	18	0.76	0.010
				232		13		
11	4-CEC	6.63	212	**159**	27	18	0.74	0.050
				194		13		
12	Dihydro-4-CEC	6.71	214	**181**	23	23	-	0.050
				141 *		14		
13	Dihydro-4-EMC	6.84	194	**117**	26	22	-	0.010
				176 *		12		
14	4-Cl-α-PPP	6.87	238	**139**	30	27	0.87	0.010
				98		25		
15	4-EMC	6.92	192	**145**	26	21	0.51	0.010
				174		12		
16	Dihydro-4-Cl-α-PPP	6.96	240	**207**	30	22	-	0.010
				115 *		30		
17	α-PVP	7.00	232	**91**	35	25	0.24	0.010
				105		21		
18	MDPV	7.16	276	**126**	30	27	0.52	0.010
				135		24		
19	Dihydro-4-MPD	7.17	208	**147**	25	22	-	0.010
				159 *		16		
20	Dihydro-MDPV	7.19	278	**217**	30	22	-	0.010
				260*		16		
21	4-MPD	7.25	206	**188**	25	13	0.45	0.010
				145		20		
22	*N*-ethylhexedrone	7.48	220	**202**	27	14	0.55	0.010
				91		22		
23	Dihydro-*N*-ethylhexedrone	7.55	222	**147**	27	23	-	0.010
				117 *		22		
24	4-F-PHP	7.90	264	**109**	35	25	0.39	0.050
				140		30		
25	4-Cl-α-PVP	7.92	266	**125**	31	21	0.26	0.050
				139		24		
26	Dihydro-4-F-PHP	8.01	266	**109**	35	25	-	0.050
				191 *		20		
27	MDPV-*d*_8_	6.84	284	**134**	33	26	-	0.020

* Only used as an additional qualifier to confirm positive metabolite findings.

**Table 2:** Calibration parameters, limit of detection and limit of quantification for all analytes in urine samples.

Analyte	LOD (ng/mL)	LOQ (ng/mL)	Intercept ± SD (*n* = 5)	Intercept ± SD (*n* = 5)	Slope ± SD (*n* = 5)	R^2^ ± SD (*n* = 5)
Mephedrone	0.22	1	1–1000	0.0023 ± 0.0079	0.1173 ± 0.0119	0.9974 ± 0.0009
Methylone	0.11	1	1–1000	−0.0105 ± 0.0037	0.1154 ± 0.0168	0.9978 ± 0.0021
Methedrone	0.37	1	1–1000	0.0013 ± 0.0075	0.0662 ± 0.0039	0.9975 ± 0.0026
Ethylone	0.23	1	1–1000	−0.0005 ± 0.0084	0.1206 ± 0.0094	0.9983 ± 0.0019
Butylone	0.29	1	1–1000	0.0032 ± 0.0028	0.0309 ± 0.0013	0.9979 ± 0.0015
Dibutylone	0.23	1	1–1000	0.0024 ± 0.0120	0.1714 ± 0.0256	0.9977 ± 0.0015
4-CEC	0.09	1	1–1000	−0.0042 ± 0.0017	0.0662 ± 0.0038	0.9984 ± 0.0013
4-Cl-α-PPP	0.30	1	1–1000	0.0079 ± 0.0193	0.2147 ± 0.0172	0.9979 ± 0.0014
N-ethylpentylone	0.26	1	1–1000	0.0104 ± 0.0083	0.1045 ± 0.0039	0.9984 ± 0.0007
4-EMC	0.11	1	1–1000	0.0068 ± 0.0035	0.1021 ± 0.0064	0.9984 ± 0.0016
α-PVP	0.13	1	1–1000	−0.0219 ± 0.0090	0.2255 ± 0.0157	0.9988 ± 0.0008
MDPV	0.35	1	1–1000	−0.0033 ± 0.0159	0.1511 ± 0.0147	0.9987 ± 0.0009
4-MPD	0.22	1	1–1000	−0.0252 ± 0.0255	0.3841 ± 0.0395	0.9960 ± 0.0039
N-ethylhexedrone	0.25	1	1–1000	−0.0040 ± 0.0141	0.1901 ± 0.0140	0.9993 ± 0.0003
4-F-PHP	0.19	1	1–1000	−0.0246 ± 0.0167	0.2849 ± 0.0234	0.9972 ± 0.0027
4-Cl-α-PVP	0.30	1	1–1000	0.0050 ± 0.0162	0.1761 ± 0.0139	0.9990 ± 0.0006
Dihydro-mephedrone	0.39	1	1–1000	0.0174 ± 0.0168	0.1426 ± 0.0212	0.9983 ± 0.0012
Dihydro-MDPV	0.43	1	1–1000	0.0224 ± 0.0104	0.0797 ± 0.0059	0.9985 ± 0.0015
Dihydro-4-Cl-α-PPP	0.49	1	1–1000	0.0292 ± 0.0183	0.1223 ± 0.0137	0.9965 ± 0.0030
Dihydro-4-EMC	0.23	1	1–1000	0.0218 ± 0.0114	0.1609 ± 0.0195	0.9984 ± 0.0009
Dihydro-*N*-ethylhexedrone	0.32	1	1–1000	0.0296 ± 0.0239	0.2455 ± 0.0189	0.9981 ± 0.0014
Dihydro-dibutylone	0.20	1	1–1000	0.0696 ± 0.0700	1.1340 ± 0.1303	0.9989 ± 0.0010
Dihydro-*N*-ethylpentylone	0.36	1	1–1000	0.0305 ± 0.0176	0.1610 ± 0.0121	0.9989 ± 0.0013
Dihydro-4-MPD	0.25	1	1–1000	0.1267 ± 0.0559	0.7330 ± 0.0820	0.9982 ± 0.0011
Dihydro-4-CEC	0.24	1	1–1000	0.0125 ± 0.0058	0.0802 ± 0.0076	0.9983 ± 0.0013
Dihydro-4-F-PHP	0.30	1	1–1000	−0.0081 ± 0.0436	0.4782 ± 0.0213	0.9988 ± 0.0011

**Table 3:** Bias and precision for urine at QC-low (30 ng/mL), QC-medium (400 ng/mL) and QC-high (800 ng/mL) concentrations.

Analyte	Nominal Conc.		Run 1 (*n* = 3)	Run 2 (*n* = 3)	Run 3 (*n* = 3)	Run 4 (*n* = 3)	Run 5 (*n* = 3)	Grand Average (*n* = 15)	Bias(%) (*n* = 15)	Within-Run (*n* = 15)	Between-Run (*n* = 15)
Mephedrone	30	CV (%)	5.6	4.4	6.8	2.9	4.7	25.7	−14.4	7.7	7.8
		Bias (%)	−2.7	−19.3	−12.5	−17.2	−16.2				
	400	CV (%)	3.6	8.8	4.9	2.5	6.2	429.5	7.4	6.9	7.0
		Bias (%)	−1.9	8.5	10.9	3.6	12.8				
	800	CV (%)	2.5	0.8	7.2	1.2	4.3	823.4	2.9	7.4	7.6
		Bias (%)	3.1	−3.4	6.5	−4.1	12.5				
Methylone	30	CV (%)	0.1	0.7	2.3	0.9	2.0	25.4	−15.5	4.7	4.9
		Bias (%)	−16.6	−19.9	−18.3	−10.4	−12.6				
	400	CV (%)	3.4	0.6	1.9	2.2	2.9	414.4	3.6	5.5	5.7
		Bias (%)	−1.4	−1.8	8.0	0.6	10.8				
	800	CV (%)	4.1	1.0	1.6	2.1	3.5	840.7	5.1	7.0	7.2
		Bias (%)	−1.3	2.9	0.0	4.3	17.3				
Methedrone	30	CV (%)	11.0	8.3	8.7	1.9	4.0	27.3	−9.0	9.6	9.7
		Bias (%)	0.1	−13.4	−0.8	−15.5	−12.3				
	400	CV (%)	3.6	3.2	5.0	2.2	7.0	386.4	−3.4	7.9	8.1
		Bias (%)	−14.9	1.4	−1.9	−8.8	3.3				
	800	CV (%)	2.0	2.0	2.5	2.4	4.4	787.0	−1.6	3.5	3.5
		Bias (%)	−1.0	−2.5	−4.6	−3.0	2.1				
Ethylone	30	CV (%)	0.9	3.7	5.3	5.7	5.5	26.3	−12.2	8.7	9.0
		Bias (%)	0.8	−14.2	−7.8	−19.2	−16.3				
	400	CV (%)	6.6	3.6	3.3	2.7	2.1	411.8	0.3	6.2	6.4
		Bias (%)	−9.7	6.7	7.1	1.9	4.5				
	800	CV (%)	4.5	0.4	1.9	0.6	2.7	800.1	0.0	2.9	3.0
		Bias (%)	−1.8	−1.6	−2.1	2.6	2.4				
Butylone	30	CV (%)	9.3	8.4	9.2	4.9	1.7	27.4	−8.7	11.0	11.3
		Bias (%)	4.2	−12.5	−13.5	−17.8	0.5				
	400	CV (%)	0.2	3.2	4.7	5.8	1.4	391.4	−2.2	9.3	9.6
		Bias (%)	−18.6	6.9	−0.7	−6.8	2.9				
	800	CV (%)	2.9	4.6	0.7	5.2	2.9	770.1	−3.7	5.7	5.8
		Bias (%)	−8.0	0.7	−9.2	−5.1	1.5				
Dibutylone	30	CV (%)	7.1	2.2	3.8	7.1	2.3	27.3	−9.1	9.9	10.2
		Bias (%)	7.8	−5.1	−16.8	−12.5	−13.0				
	400	CV (%)	1.8	13.9	7.7	4.0	3.1	433.6	8.4	8.6	8.6
		Bias (%)	8.0	12.0	0.9	4.5	16.5				
	800	CV (%)	4.6	11.0	10.1	7.6	2.3	816.7	2.1	7.8	7.8
		Bias (%)	−1.7	−0.5	−0.6	9.1	2.8				
4-CEC	30	CV (%)	8.4	1.6	3.7	2.2	1.0	25.0	−16.6	4.3	4.4
		Bias (%)	−14.6	−17.2	−18.6	−12.6	−19.2				
	400	CV (%)	1.8	5.1	1.3	3.3	5.3	419.3	4.8	7.0	7.2
		Bias (%)	−8.6	3.1	8.3	5.3	11.6				
	800	CV (%)	2.4	2.9	1.1	5.3	0.6	803.5	0.4	4.0	4.0
		Bias (%)	−0.8	−3.2	−1.7	4.0	3.4				
4-Cl-α-PPP	30	CV (%)	5.1	9.2	8.5	3.6	2.1	28.3	−5.8	12.2	12.5
		Bias (%)	9.3	−8.8	4.7	−10.7	−18.3				
	400	CV (%)	3.4	1.5	1.8	3.5	2.2	420.1	7.5	7.5	7.8
		Bias (%)	−12.3	5.2	10.4	8.1	7.8				
	800	CV (%)	4.0	1.4	1.0	0.5	2.1	815.4	1.9	4.9	5.1
		Bias (%)	6.2	−5.5	0.5	2.7	7.1				
*N*-ethylpentylone	30	CV (%)	0.6	6.0	3.1	2.0	8.8	28.2	−6.2	11.5	11.8
		Bias (%)	11.6	−12.6	2.7	−12.1	−14.5				
	400	CV (%)	6.8	5.7	2.2	3.3	3.3	423.2	5.8	5.3	5.4
		Bias (%)	1.5	0.3	11.1	6.3	8.4				
	800	CV (%)	2.2	3.7	1.9	3.8	1.5	779.4	−2.6	3.7	3.7
		Bias (%)	−4.0	−6.1	−3.9	−0.3	0.9				
4-EMC	30	CV (%)	5.8	5.0	2.8	3.3	7.5	27.4	−8.5	10.5	10.8
		Bias (%)	−3.5	−7.1	3.9	−19.7	−14.6				
	400	CV (%)	3.5	5.2	2.9	4.7	4.6	402.4	0.6	6.6	6.7
		Bias (%)	−12.3	2.2	3.7	2.6	2.6				
	800	CV (%)	2.2	3.1	0.9	5.6	2.0	811.5	1.4	3.2	3.2
		Bias (%)	3.3	1.7	−1.4	1.8	2.5				
α-PVP	30	CV (%)	1.2	0.5	5.9	1.6	0.7	24.9	−16.9	4.5	4.6
		Bias (%)	−9.7	−19.5	−17.5	−18.2	−16.9				
	400	CV (%)	8.3	6.0	3.4	6.2	2.0	401.0	0.3	6.0	6.1
		Bias (%)	−7.5	−2.6	4.9	0.9	3.0				
	800	CV (%)	1.7	3.8	0.8	2.7	4.9	812.3	1.5	4.4	4.5
		Bias (%)	0.9	−3.6	0.2	4.2	5.8				
MDPV	30	CV (%)	10.7	10.2	4.5	1.8	5.6	26.6	−11.2	7.9	12.5
		Bias (%)	5.0	−10.9	−18.1	−15.4	−16.7				
	400	CV (%)	0.3	5.3	4.4	4.5	5.7	415.1	3.8	4.5	5.0
		Bias (%)	−0.6	7.0	6.0	0.6	5.9				
	800	CV (%)	1.7	5.3	1.3	2.6	4.5	817.9	2.2	3.5	5.4
		Bias (%)	−3.6	1.2	3.5	0.9	9.2				
4-MPD	30	CV (%)	3.0	1.9	3.1	5.5	4.3	27.9	−7.0	12.9	13.4
		Bias (%)	9.8	5.2	−7.8	−17.9	−18.6				
	400	CV (%)	2.0	6.3	0.4	3.2	1.7	406.7	1.7	5.8	5.9
		Bias (%)	−5.7	−2.8	4.5	1.1	8.7				
	800	CV (%)	8.5	0.6	1.4	1.9	2.4	809.5	1.2	4.9	4.9
		Bias (%)	9.3	−0.7	0.1	−2.3	2.7				
*N*-ethylhexedrone	30	CV (%)	0.6	2.0	2.0	3.3	0.2	26.8	−10.7	8.0	8.3
		Bias (%)	−1.4	−5.3	−7.4	−18.8	−17.5				
	400	CV (%)	2.8	4.5	1.0	1.6	0.8	409.4	2.4	3.1	3.1
		Bias (%)	−0.9	0.2	2.3	5.6	3.6				
	800	CV (%)	1.6	0.9	1.9	1.3	4.1	824.1	3.0	4.6	4.7
		Bias (%)	8.3	−3.1	0.7	3.7	7.2				
4-F-PHP	30	CV (%)	4.6	0.9	2.9	0.5	5.0	25.8	−14.2	4.5	4.6
		Bias (%)	−8.3	−17.0	−13.8	−16.9	−13.0				
	400	CV (%)	1.8	5.0	2.2	3.3	5.0	388.8	−2.8	6.9	7.1
		Bias (%)	−10.1	−1.7	2.1	−10.0	3.2				
	800	CV (%)	2.4	2.2	3.7	3.5	1.8	788.9	−1.4	6.0	6.2
		Bias (%)	−1.4	−3.6	2.8	−9.6	4.8				
4-Cl-α-PVP	30	CV (%)	6.0	8.4	2.3	0.9	1.6	25.1	−16.2	9.0	9.3
		Bias (%)	−0.3	−18.2	−17.6	−19.7	−19.9				
	400	CV (%)	4.4	2.2	1.2	2.3	0.4	397.6	−0.6	6.6	6.8
		Bias (%)	−11.5	0.1	−1.7	−2.3	8.8				
	800	CV (%)	0.1	2.0	1.0	0.7	2.0	785.8	−1.8	4.3	4.4
		Bias (%)	−1.4	−1.4	−5.9	−4.8	4.8				
Dihydro-mephedrone	30	CV (%)	0.9	8.8	5.9	5.4	7.8	31.5	5.1	9.2	9.4
		Bias (%)	16.0	−0.1	13.3	−4.2	4.5				
	400	CV (%)	6.1	7.3	3.2	0.8	0.4	428.6	7.1	5.8	5.9
		Bias (%)	7.1	13.5	10.3	1.6	3.2				
	800	CV (%)	3.1	4.5	2.9	0.9	2.2	767.0	−4.1	3.1	3.1
		Bias (%)	7.1	13.5	10.3	1.6	3.2				
Dihydro-MDPV	30	CV (%)	3.6	8.6	12.3	4.9	8.0	30.5	1.7	10.5	10.6
		Bias (%)	17.2	6.7	−0.6	−5.3	−4.4				
	400	CV (%)	6.9	4.4	6.7	2.2	1.5	410.7	2.7	5.1	5.1
		Bias (%)	−1.5	8.5	8.5	2.7	1.0				
	800	CV (%)	0.6	1.7	5.4	1.8	3.3	752.1	−6.0	5.0	5.1
		Bias (%)	−6.5	−9.9	−5.2	−8.9	0.4				
Dihydro-4-Cl-α-PPP	30	CV (%)	10.3	7.5	10.5	8.4	3.7	28.1	−6.4	8.5	8.5
		Bias (%)	13	−5.7	−3.1	−11.2	−10.7				
	400	CV (%)	0.5	5.8	5.3	3.6	1.2	429.9	7.5	5.1	5.2
		Bias (%)	−0.4	7.6	10.1	5.7	11.8				
	800	CV (%)	5.5	5.0	6.7	7.6	8.7	769.9	−3.8	7.8	7.9
		Bias (%)	−8.4	−2.7	−10.5	−1.6	2.8				
Dihydro-4-EMC	30	CV (%)	3.2	9.3	4.0	7.9	9.3	31.6	5.2	9.9	10.1
		Bias (%)	18.0	3.8	12.2	−6.0	2.4				
	400	CV (%)	3.1	1.7	2.1	3.8	1.7	438.1	9.5	6.9	7.1
		Bias (%)	−6.0	7.8	14.1	10.8	15.7				
	800	CV (%)	6.9	0.3	2.0	2.6	0.7	796.8	−0.4	4.1	4.2
		Bias (%)	−3.3	−3.9	−2.0	1.6	4.6				
Dihydro-*N*-ethylhexedrone	30	CV (%)	2.0	3.6	2.6	0.6	3.8	29.5	−1.6	5.1	5.3
		Bias (%)	5.0	1.4	−2.3	−1.3	−8.6				
	400	CV (%)	1.4	3.7	1.4	1.2	2.1	435.2	8.8	4.7	4.9
		Bias (%)	−1.8	7.8	10.6	11.5	12.3				
	800	CV (%)	3.5	1.9	2.6	4.1	4.0	801.9	0.2	5.1	5.2
		Bias (%)	−1.4	−7.0	2.9	3.0	3.1				
Dihydro-dibutylone	30	CV (%)	3.3	3.9	7.7	8.9	4.6	29.4	−1.9	8.2	8.3
		Bias (%)	−2.1	−6.2	9.1	−5.3	−4.9				
	400	CV (%)	3.6	2.2	1.0	2.0	1.6	427.4	6.8	7.9	8.2
		Bias (%)	−8.5	12.7	13.7	11.0	0.3				
	800	CV (%)	1.0	1.2	0.2	1.0	1.7	800.1	0.0	2.9	3.0
		Bias (%)	1.3	−4.1	−1.7	2.7	2.3				
Dihydro-*N*-ethylpentylone	30	CV (%)	8.7	7.8	2.5	7.9	2.2	29.1	−2.9	8.3	8.4
		Bias (%)	7.4	−9.3	2.0	−2.1	−8.9				
	400	CV (%)	4.6	0.4	3.2	7.0	2.9	430.5	7.6	6.7	6.9
		Bias (%)	−4.9	5.3	7.7	12.8	13.0				
	800	CV (%)	0.3	1.5	4.6	2.6	2.3	794.3	−0.7	6.7	7.0
		Bias (%)	−11.9	−0.8	−4.2	7.5	2.1				
Dihydro-4-MPD	30	CV (%)	2.8	4.5	6.0	5.0	2.7	31.5	5.2	7.0	7.1
		Bias (%)	18.3	3.0	3.6	−1.4	6.6				
	400	CV (%)	1.4	8.1	1.6	7.2	9.6	414.2	3.5	9.9	10.1
		Bias (%)	−9.4	−1.7	3.5	5.3	15.7				
	800	CV (%)	0.1	1.4	3.6	3.9	5.7	801.1	0.1	6.1	6.3
		Bias (%)	−1.4	−8.3	2.8	0.7	6.4				
Dihydro-4-CEC	30	CV (%)	6.8	6.8	9.4	3.0	5.2	31.0	3.2	8.3	8.4
		Bias (%)	15.3	0.3	7.6	−3.0	0.0				
	400	CV (%)	1.7	2.1	3.5	1.7	4.0	432.8	8.2	7.1	7.3
		Bias (%)	6.0	12.9	13.0	12.7	−4.4				
	800	CV (%)	0.0	1.3	1.3	2.0	1.5	795.6	−0.5	2.2	2.2
		Bias (%)	1.8	−2.3	−2.2	1.4	−0.7				
Dihydro-4-F-PHP	30	CV (%)	10.2	6.9	1.2	0.2	1.0	25.8	7.2	7.2	7.4
		Bias (%)	−3.0	−17.8	−15.7	−12.0	−17.2				
	400	CV (%)	11.6	2.4	15.0	8.3	0.9	410.1	2.5	10.2	10.4
		Bias (%)	−11.6	4.1	−1.9	6.4	10.9				
	800	CV (%)	0.4	5.7	4.8	4.0	4.7	801.1	0.1	5.1	5.1
		Bias (%)	0.0	−5.1	0.5	0.5	4.8				

**Table 4:** Processed sample stability of SCts in urine after 24, 48 and 72 h of storage on autosampler (10 °C) at QC-low (30 ng/mL) and QC-high (800 ng/mL). (*n* = 3).

	24 h		48 h		72 h	
	QC-Low	QC-High	QC-Low	QC-High	QC-Low	QC-High
**Analyte**	% Loss (RSD)	% Loss (RSD)	% Loss (RSD)	% Loss (RSD)	% Loss (RSD)	% Loss (RSD)
Mephedrone	−1.1	1.0	−2.9	0.4	−2.0	−1.1
	(1.0)	(3.6)	(1.8)	(2.3)	(2.7)	(1.0)
Methylone	9.2	0.0	−8.3	−5.1	−8.1	9.2
	(5.2)	(3.4)	(7.0)	(2.4)	(12.6)	(5.2)
Methedrone	−6.2	−11.6	−8.5	−11.9	−12.3	−6.2
	(18.3)	(2.2)	(15.6)	(4.9)	(14.6)	(18.3)
Ethylone	−7.1	1.7	−12.2	−1.7	−14.5	−0.4
	(6.2)	(2.4)	(6.6)	(4.0)	(6.8)	(4.5)
Butylone	3.8	−5.9	−14.2	−13.0	−12.7	−11.7
	(2.6)	(8.8)	(17.0)	(5.4)	(8.9)	(3.1)
Dibutylone	5.7	0.2	4.1	−0.3	−6.5	−1.3
	(3.5)	(2.9)	(6.5)	(1.8)	(5.2)	(1.1)
4-CEC	0.3	−8.5	5.1	−2.5	2.0	−3.5
	(8.1)	(8.8)	(14.3)	(9.8)	(4.3)	(4.4)
4-Cl-α-PPP	−2.2	−5.5	3.2	−2.2	1.4	−4.8
	(6.6)	(3.1)	(8.4)	(1.2)	(8.9)	(2.2)
*N*-ethylpentylone	0.1	−9.2	−8.5	−4.3	−2.1	−11.4
	(9.6)	(8.5)	(10.6)	(2.0)	(7.2)	(0.6)
4-EMC	−9.4	−6.8	−6.6	2.1	−7.0	−1.2
	(14.4)	(9.8)	(14.9)	(2.0)	(6.3)	(7.8)
α-PVP	−3.2	−10.8	9.9	−5.6	0.4	−13.3
	(4.8)	(3.3)	(14.3)	(1.5)	(3.1)	(2.1)
MDPV	3.6	−8.8	5.6	0.3	1.7	−1.2
	(8.0)	(17.3)	(8.9)	(3.7)	(8.0)	(7.4)
4-MPD	1.5	−0.7	−3.0	−1.0	−0.5	−10.5
	(6.9)	(5.3)	(13.9)	(2.8)	(5.5)	(6.6)
*N*-ethylhexedrone	−3.6	−6.5	−5.9	1.8	−5.8	−5.7
	(13.1)	(2.7)	(12.2)	(1.0)	(6.1)	(2.1)
4-F-PHP	−7.7	−9.0	−6.5	−2.4	−13.8	−11.0
	(12.8)	(4.8)	(3.4)	(0.3)	(3.9)	(0.6)
4-Cl-α-PVP	−10.1	−7.0	−12.6	−4.8	−16.1	−11.8
	(17.4)	(14.4)	(12.0)	(2.9)	(7.4)	(3.0)
Dihydro-mephedrone	−12.5	−5.4	−17.3	−4.2	−15.1	−2.4
	(11.5)	(5.0)	(15.0)	(5.0)	(12.5)	(1.9)
Dihydro-MDPV	−13.0	−10.3	−7.7	1.3	−18.0	−5.4
	(15.0)	(8.3)	(11.9)	(5.3)	(6.3)	(8.5)
Dihydro-4-Cl-α-PPP	8.8	−3.4	10.8	−4.0	6.8	−6.6
	(15.6)	(2.3)	(10.3)	(4.3)	(14.8)	(3.1)
Dihydro-4-EMC	−1.9	−5.7	−0.7	3.2	−17.0	−3.9
	(3.3)	(2.7)	(17.6)	(1.7)	(6.3)	(2.3)
Dihydro-*N*-ethylhexedrone	−3.9	−3.8	2.0	6.6	−1.6	−2.1
	(11.1)	(6.1)	(16.5)	(3.0)	(3.3)	(0.9)
Dihydro-dibutylone	−3.8	−7.9	4.8	−6.5	−8.6	−8.0
	(11.8)	(6.8)	(13.9)	(2.0)	(21.0)	(1.8)
Dihydro-*N*-ethylpentylone	−3.0	−9.1	−5.4	−5.6	−1.7	−9.4
	(2.1)	(3.9)	(6.2)	(3.0)	(5.9)	(2.5)
Dihydro-4-MPD	3.9	−5.3	0.7	−2.2	1.6	−7.9
	(13.8)	(10.9)	(15.1)	(2.9)	(8.5)	(1.1)
Dihydro-4-CEC	−3.2	−4.7	5.5	−1.8	−3.0	−4.1
	(11.1)	(5.3)	(4.0)	(2.8)	(6.1)	(2.0)
Dihydro-4-F-PHP	−8.8	−12.7	−3.3	−9.2	−5.4	−10.9
	(6.3)	(13.0)	(11.8)	(7.7)	(12.5)	(1.9)

**Table 5:** Findings from analysis of authentic urine samples.

Sample No.	Detected Target Analytes (ng/mL)	Other Stimulant Findings (Qualitative Screening)
19	Dihydro-mephedrone (metabolite): 172	Methamphetamine (+) *
34	*N*-ethylpentylone: 52; dihydro-*N*-ethylpentylone (metabolite); 1378	Methamphetamine (+++) *
49	Mephedrone: 1537; dihydro-mephedrone (metabolite): >5000	Methamphetamine (+++); MDMA (+) *

* The number of (+) indicates relative abundance of the respective analyte as estimated from signal intensities in the chromatograms.

## Data Availability

Data is contained within the article and supplementary material.

## Supplementary Materials

The following supporting information can be downloaded at: https://www.mdpi.com/article/10.3390/ph15050510/s1, Figure S1: Matrix effects of SCts and metabolites in urine at 30 and 800 ng/mL. The error bars show RSD (n = 5), Figure S2: Confirmatory results for an authentic urine sample (no. 19) testing positive for dihydro-mephedrone (left); and a QC sample (400 ng/mL; right), Figure S3: Confirmatory results for an authentic urine sample (no. 34) testing positive for dihydro-N-ethylpentylone (left); and a QC sample (400 ng/mL; right), Figure S4: Confirmatory results for an authentic urine sample (no. 49) testing positive for dihydro-mephedrone (left); and a QC sample (400 ng/mL; right), Figure S5: Representative MRM chromatograms of ethylone (A) and butylone (B), Figure S6: Reduction of mephedrone to dihydro-mephedrone, Table S1: Group A and B of controls comprising of common drugs (n = 196) at a concentration of 10 or 100 ng/mL (depending on the drug), Table S2: Calculated ion ratio tolerance between less intense peak area to that of more intense peak area and that which was established by the spiked QC samples for metabolites’ positive findings, Table S3: Maximum permitted tolerance for ion ratio, Table S4: Storage and shipment conditions for positive urine samples, Table S5: Calculated ion ratio tolerance between less intense peak area to that of more intense and that was established by the spiked QC samples for metabolites positive findings, Table S6: Maximum permitted tolerance for ion ratio.
