# Supplementary material for: A Quantitative LC–MS/MS Method for the Detection of 16 Synthetic Cathinones and 10 Metabolites and Its Application to Suspicious Clinical and Forensic Urine Samples
Source: Pharmaceuticals (Basel). 2022 Apr 22;15(5):510. doi: 10.3390/ph15050510 (PMC9145040; doi:10.3390/ph15050510)
Supplement: Supplementary file 1 [file pharmaceuticals-15-00510-s001.zip › pharmaceuticals-1671806-supplementary.pdf]

Supplementary Materials:

A Quantitative LC-MS/MS Method for the Detection of 26 Synthetic Cathinones and Metabolites and Its Application to Suspicious Clinical and Forensic URINE samples

Abdulaziz A. Aldubayyan [1,2], Erika Castrignanò [1], Simon Elliott [1,3] and Vincenzo Abbate [1,*]

[1] Department of Analytical, Environmental & Forensic Sciences, Faculty of Life Sciences & Medicine, King's College London, London SE1 9NH, UK; abdulaziz.aldubayyan@kcl.ac.uk or aaldubayyan@pscc.med.sa (A.A.A.); erika.castrignano@kcl.ac.uk (E.C.); simon.elliott@kcl.ac.uk (S.E.)

[2] Department of Toxicology, Central Military Laboratory and Blood Bank, Prince Sultan Military Medical City, Riyadh 12233, Saudi Arabia

[3] Elliott Forensic Consulting, Birmingham B16 9HN, UK

* Correspondence: vincenzo.abbate@kcl.ac.uk

Content of Tables **Page**

Table S1. Calibration parameters, limit of detection, and limit of quantification for all analytes in urine samples.. 4

Table S2. Bias and precision for urine at QC-low (30 ng/mL), QC-medium (400 ng/mL) and QC-high (800 ng/mL) concentrations. 5

Table S3. Group A and B of controls comprising of common drugs (n=196) at a concentration of 10 or 100 ng/mL (depending on the drug). 12

Table S4. Processed sample stability of SCt in urine after 24, 48 and 72 h of storage on autosampler (10°C) at QC-low (30 ng/mL) and QC-high (800 ng/mL). 16

Table S5. Calculated ion ratio tolerance between less intense peak area to that of more intense and that was established by the spiked QC samples for metabolites positive findings..........................18

Table S6. Maximum permitted tolerance for ion ratio 18

Content of Figures **Page**

Figure S1. Matrix effects of SCt and metabolites in urine at 30 and 800 ng/mL. The error bars show RSD (n = 5). 20

Figure S2. Confirmatory results for an authentic urine sample (No. 19) testing positive for dihydro-mephedrone (left); and a QC sample (400 ng/mL; right) 21

Figure S3. Confirmatory results for an authentic urine sample (No. 34) testing positive for dihydro-*N*-ethylpentylone (left); and a QC sample (400 ng/mL; right) 22

Figure S4. Confirmatory results for an authentic urine sample (No. 49) testing positive for dihydro-mephedrone (left); and a QC sample (400 ng/mL; right) 23

Figure S5. Representative MRM chromatograms of ethylone (A) and butylone (B) 24

Figure S6. Reduction of mephedrone to dihydro-mephedrone 25

Table S1. Calibration parameters, limit of detection, and limit of quantification for all analytes in urine samples.

Analyte	LOD (ng/mL)	LOQ (ng/mL)	Intercept ± SD (n=5)	Intercept ± SD (n=5)	Slope ± SD (n=5)	R^2 ± SD (n=5)
Mephedrone	0.22	1	1–1000	0.0023 ± 0.0079	0.1173 ± 0.0119	0.9974 ± 0.0009
Methylone	0.11	1	1–1000	-0.0105 ± 0.0037	0.1154 ± 0.0168	0.9978 ± 0.0021
Methedrone	0.37	1	1–1000	0.0013 ± 0.0075	0.0662 ± 0.0039	0.9975 ± 0.0026
Ethylone	0.23	1	1–1000	-0.0005 ± 0.0084	0.1206 ± 0.0094	0.9983 ± 0.0019
Butylone	0.29	1	1–1000	0.0032 ± 0.0028	0.0309 ± 0.0013	0.9979 ± 0.0015
Dibutylone	0.23	1	1–1000	0.0024 ± 0.0120	0.1714 ± 0.0256	0.9977 ± 0.0015
4-CEC	0.09	1	1–1000	-0.0042 ± 0.0017	0.0662 ± 0.0038	0.9984 ± 0.0013
4-Cl-α-PPP	0.30	1	1–1000	0.0079 ± 0.0193	0.2147 ± 0.0172	0.9979 ± 0.0014
N-ethylpentylone	0.26	1	1–1000	0.0104 ± 0.0083	0.1045 ± 0.0039	0.9984 ± 0.0007
4-EMC	0.11	1	1–1000	0.0068 ± 0.0035	0.1021 ± 0.0064	0.9984 ± 0.0016
α -PVP	0.13	1	1–1000	-0.0219 ± 0.0090	0.2255 ± 0.0157	0.9988 ± 0.0008
MDPV	0.35	1	1–1000	-0.0033 ± 0.0159	0.1511 ± 0.0147	0.9987 ± 0.0009
4-MPD	0.22	1	1–1000	-0.0252 ± 0.0255	0.3841 ± 0.0395	0.9960 ± 0.0039
N-ethylhexedrone	0.25	1	1–1000	-0.0040 ± 0.0141	0.1901 ± 0.0140	0.9993 ± 0.0003
4-F-PHP	0.19	1	1–1000	-0.0246 ± 0.0167	0.2849 ± 0.0234	0.9972 ± 0.0027
4-Cl-α-PVP	0.30	1	1–1000	0.0050 ± 0.0162	0.1761 ± 0.0139	0.9990 ± 0.0006
Dihydro-mephedrone	0.39	1	1–1000	0.0174 ± 0.0168	0.1426 ± 0.0212	0.9983 ± 0.0012
Dihydro-MDPV	0.43	1	1–1000	0.0224 ± 0.0104	0.0797 ± 0.0059	0.9985 ± 0.0015
Dihydro-4-Cl-α-PPP	0.49	1	1–1000	0.0292 ± 0.0183	0.1223 ± 0.0137	0.9965 ± 0.0030
Dihydro-4-EMC	0.23	1	1–1000	0.0218 ± 0.0114	0.1609 ± 0.0195	0.9984 ± 0.0009
Dihydro-*N*-ethylhexedrone	0.32	1	1–1000	0.0296 ± 0.0239	0.2455 ± 0.0189	0.9981 ± 0.0014
Dihydro-dibutylone	0.20	1	1–1000	0.0696 ± 0.0700	1.1340 ± 0.1303	0.9989 ± 0.0010
Dihydro-*N*-ethylpentylone	0.36	1	1–1000	0.0305 ± 0.0176	0.1610 ± 0.0121	0.9989 ± 0.0013
Dihydro-4-MPD	0.25	1	1–1000	0.1267 ± 0.0559	0.7330 ± 0.0820	0.9982 ± 0.0011
Dihydro-4-CEC	0.24	1	1–1000	0.0125 ± 0.0058	0.0802 ± 0.0076	0.9983 ± 0.0013
Dihydro-4-F-PHP	0.30	1	1–1000	-0.0081 ± 0.0436	0.4782 ± 0.0213	0.9988 ± 0.0011

Table S2. Bias and precision for urine at QC-low (30 ng/mL), QC-medium (400 ng/mL) and QC-high (800 ng/mL) concentrations.

Analyte	Nominal concentration (ng/mL)		Run 1 (n=3)	Run 2 (n=3)	Run 3 (n=3)	Run 4 (n=3)	Run 5 (n=3)	Grand ave. (n=15)	Bias (%) (n=15)	CV (%) within run (n=15)	CV (%) between run (n=15)
Mephedrone	30	CV (%)<br>Bias (%)	5.6<br>-2.7	4.4<br>-19.3	6.8<br>-12.5	2.9<br>-17.2	4.7<br>-16.2	25.7	-14.4	7.7	7.8
	400	CV (%)<br>Bias (%)	3.6<br>-1.9	8.8<br>8.5	4.9<br>10.9	2.5<br>3.6	6.2<br>12.8	429.5	7.4	6.9	7.0
	800	CV (%)<br>Bias (%)	2.5<br>3.1	0.8<br>-3.4	7.2<br>6.5	1.2<br>-4.1	4.3<br>12.5	823.4	2.9	7.4	7.6
Methylone	30	CV (%)<br>Bias (%)	0.1<br>-16.6	0.7<br>-19.9	2.3<br>-18.3	0.9<br>-10.4	2.0<br>-12.6	25.4	-15.5	4.7	4.9
	400	CV (%)<br>Bias (%)	3.4<br>-1.4	0.6<br>-1.8	1.9<br>8.0	2.2<br>0.6	2.9<br>10.8	414.4	3.6	5.5	5.7
	800	CV (%)<br>Bias (%)	4.1<br>-1.3	1.0<br>2.9	1.6<br>0.0	2.1<br>4.3	3.5<br>17.3	840.7	5.1	7.0	7.2
Methedrone	30	CV (%)<br>Bias (%)	11.0<br>0.1	8.3<br>-13.4	8.7<br>-0.8	1.9<br>-15.5	4.0<br>-12.3	27.3	-9.0	9.6	9.7
	400	CV (%)<br>Bias (%)	3.6<br>-14.9	3.2<br>1.4	5.0<br>-1.9	2.2<br>-8.8	7.0<br>3.3	386.4	-3.4	7.9	8.1

	800	CV (%)<br>Bias (%)	2.0<br>-1.0	2.0<br>-2.5	2.5<br>-4.6	2.4<br>-3.0	4.4<br>2.1	787.0	-1.6	3.5	3.5
Ethylone	30	CV (%)<br>Bias (%)	0.9<br>0.8	3.7<br>-14.2	5.3<br>-7.8	5.7<br>-19.2	5.5<br>-16.3	26.3	-12.2	8.7	9.0
	400	CV (%)<br>Bias (%)	6.6<br>-9.7	3.6<br>6.7	3.3<br>7.1	2.7<br>1.9	2.1<br>4.5	411.8	0.26	6.2	6.4
	800	CV (%)<br>Bias (%)	4.5<br>-1.8	0.4<br>-1.6	1.9<br>-2.1	0.6<br>2.6	2.7<br>2.4	800.1	0.0	2.9	3.0
Butylone	30	CV (%)<br>Bias (%)	9.3<br>4.2	8.4<br>-12.5	9.2<br>-13.5	4.9<br>-17.8	1.7<br>0.5	27.4	-8.7	11.0	11.3
	400	CV (%)<br>Bias (%)	0.2<br>-18.6	3.2<br>6.9	4.7<br>-0.7	5.8<br>-6.8	1.4<br>2.9	391.4	-2.2	9.3	9.6
	800	CV (%)<br>Bias (%)	2.9<br>-8.0	4.6<br>0.7	0.7<br>-9.2	5.2<br>-5.1	2.9<br>1.5	770.1	-3.7	5.7	5.8
Dibutylone	30	CV (%)<br>Bias (%)	7.1<br>7.8	2.2<br>-5.1	3.8<br>-16.8	7.1<br>-12.5	2.3<br>-13.0	27.3	-9.1	9.9	10.2
	400	CV (%)<br>Bias (%)	1.8<br>8.0	13.9<br>12.0	7.7<br>0.9	4.0<br>4.5	3.1<br>16.5	433.6	8.4	8.6	8.6
	800	CV (%)<br>Bias (%)	4.6<br>-1.7	11.0<br>-0.5	10.1<br>-0.6	7.6<br>9.1	2.3<br>2.8	816.7	2.1	7.8	7.8
4-CEC	30	CV (%)<br>Bias (%)	8.4<br>-14.6	1.6<br>-17.2	3.7<br>-18.6	2.2<br>-12.6	1.0<br>-19.2	25.0	-16.6	4.3	4.4

	400	CV (%)<br>Bias (%)	1.8<br>-8.6	5.1<br>3.1	1.3<br>8.3	3.3<br>5.3	5.3<br>11.6	419.3	4.8	7.0	7.2
	800	CV (%)<br>Bias (%)	2.4<br>-0.8	2.9<br>-3.2	1.1<br>-1.7	5.3<br>4.0	0.6<br>3.4	803.5	0.4	4.0	4.0
4-Cl- α -PPP	30	CV (%)<br>Bias (%)	5.1<br>9.3	9.2<br>-8.8	8.5<br>4.7	3.6<br>-10.7	2.1<br>-18.3	28.3	-5.8	12.2	12.5
	400	CV (%)<br>Bias (%)	3.4<br>-12.3	1.5<br>5.2	1.8<br>10.4	3.5<br>8.1	2.2<br>7.8	420.1	7.5	7.5	7.8
	800	CV (%)<br>Bias (%)	4.0<br>6.2	1.4<br>-5.5	1.0<br>0.5	0.5<br>2.7	2.1<br>7.1	815.4	1.9	4.9	5.1
N-ethylpentylone	30	CV (%)<br>Bias (%)	0.6<br>11.6	6.0<br>-12.6	3.1<br>2.7	2.0<br>-12.1	8.8<br>-14.5	28.2	-6.2	11.5	11.8
	400	CV (%)<br>Bias (%)	6.8<br>1.5	5.7<br>0.3	2.2<br>11.1	3.3<br>6.3	3.3<br>8.4	423.2	5.8	5.3	5.4
	800	CV (%)<br>Bias (%)	2.2<br>-4.0	3.7<br>-6.1	1.9<br>-3.9	3.8<br>-0.3	1.5<br>0.9	779.4	-2.6	3.7	3.7
4-EMC	30	CV (%)<br>Bias (%)	5.8<br>-3.5	5.0<br>-7.1	2.8<br>3.9	3.3<br>-19.7	7.5<br>-14.6	27.4	-8.5	10.5	10.8
	400	CV (%)<br>Bias (%)	3.5<br>-12.3	5.2<br>2.2	2.9<br>3.7	4.7<br>2.6	4.6<br>2.6	402.4	0.6	6.6	6.7
	800	CV (%)<br>Bias (%)	2.2<br>3.3	3.1<br>1.7	0.9<br>-1.4	5.6<br>1.8	2.0<br>2.5	811.5	1.4	3.2	3.2

α -PVP	30	CV (%)	1.2	0.5	5.9	1.6	0.7	24.9	-16.9	4.5	4.6
		Bias (%)	-9.7	-19.5	-17.5	-18.2	-16.9				
	400	CV (%)	8.3	6.0	3.4	6.2	2.0	401.0	0.3	6.0	6.1
		Bias (%)	-7.5	-2.6	4.9	0.9	3.0				
	800	CV (%)	1.7	3.8	0.8	2.7	4.9	812.3	1.5	4.4	4.5
		Bias (%)	0.9	-3.6	0.2	4.2	5.8				
4-MPD	30	CV (%)	3.0	1.9	3.1	5.5	4.3	27.9	-7.0	12.9	13.4
		Bias (%)	9.8	5.2	-7.8	-17.9	-18.6				
	400	CV (%)	2.0	6.3	0.4	3.2	1.7	406.7	1.7	5.8	5.9
		Bias (%)	-5.7	-2.8	4.5	1.1	8.7				
	800	CV (%)	8.5	0.6	1.4	1.9	2.4	809.5	1.2	4.9	4.9
		Bias (%)	9.3	-0.7	0.1	-2.3	2.7				
N-ethylhexedrone	30	CV (%)	0.6	2.0	2.0	3.3	0.2	26.8	-10.7	8.0	8.3
		Bias (%)	-1.4	-5.3	-7.4	-18.8	-17.5				
	400	CV (%)	2.8	4.5	1.0	1.6	0.8	409.4	2.4	3.1	3.1
		Bias (%)	-0.9	0.2	2.3	5.6	3.6				
	800	CV (%)	1.6	0.9	1.9	1.3	4.1	824.1	3.0	4.6	4.7
		Bias (%)	8.3	-3.1	0.7	3.7	7.2				
4-F-PHP	30	CV (%)	4.6	0.9	2.9	0.5	5.0	25.8	-14.2	4.5	4.6
		Bias (%)	-8.3	-17.0	-13.8	-16.9	-13.0				
	400	CV (%)	1.8	5.0	2.2	3.3	5.0	388.8	-2.8	6.9	7.1
		Bias (%)	-10.1	-1.7	2.1	-10.0	3.2				

	800	CV (%)	2.4	2.2	3.7	3.5	1.8			6.0	6.2
		Bias (%)	-1.4	-3.6	2.8	-9.6	4.8	788.9	-1.4		
4-Cl- α -PVP	30	CV (%)	6.0	8.4	2.3	0.9	1.6			9.0	9.3
		Bias (%)	-0.3	-18.2	-17.6	-19.7	-19.9	25.1	-16.2		
	400	CV (%)	4.4	2.2	1.2	2.3	0.4	397.6	-0.6	6.6	6.8
		Bias (%)	-11.5	0.1	-1.7	-2.3	8.8				
	800	CV (%)	0.1	2.0	1.0	0.7	2.0			4.3	4.4
		Bias (%)	-1.4	-1.4	-5.9	-4.8	4.8	785.8	-1.8		
Dihydro-mephedrone	30	CV (%)	0.9	8.8	5.9	5.4	7.8			9.2	9.4
		Bias (%)	16.0	-0.1	13.3	-4.2	4.5	31.5	5.1		
	400	CV (%)	6.1	7.3	3.2	0.8	0.4	428.6	7.1	5.8	5.9
		Bias (%)	7.1	13.5	10.3	1.6	3.2				
	800	CV (%)	3.1	4.5	2.9	0.9	2.2			3.1	3.1
		Bias (%)	-4.6	-7.0	-2.5	-4.4	-2.3	767.0	-4.1		
Dihydro-MDPV	30	CV (%)	3.6	8.6	12.3	4.9	8.0			10.5	10.6
		Bias (%)	17.2	6.7	-0.6	-5.3	-4.4	30.5	1.7		
	400	CV (%)	6.9	4.4	6.7	2.2	1.5	410.7	2.7	5.1	5.1
		Bias (%)	-1.5	8.5	8.5	2.7	1.0				
	800	CV (%)	0.6	1.7	5.4	1.8	3.3			5.0	5.1
		Bias (%)	-6.5	-9.9	-5.2	-8.9	0.4	752.1	-6.0		
Dihydro-4-Cl- α -PPP	30	CV (%)	10.3	7.5	10.5	8.4	3.7				8.5
		Bias (%)	1.3	-5.7	-3.1	-11.2	-10.7	28.1	-6.4	8.5	
	400	CV (%)	0.5	5.8	5.3	3.6	1.2			5.1	5.2
		Bias (%)	-0.4	7.6	10.1	5.7	11.8	429.9	7.5		

	800	CV (%)	5.5	5.0	6.7	7.6	8.7	769.9	-3.8	7.8	7.9
		Bias (%)	-8.4	-2.7	-10.5	-1.6	2.8				
Dihydro-4-EMC	30	CV (%)	3.2	9.3	4.0	7.9	9.3	31.6	5.2	9.9	10.1
		Bias (%)	18.0	3.8	12.2	-6.0	2.4				
	400	CV (%)	3.1	1.7	2.1	3.8	1.7	438.1	9.5	6.9	7.1
		Bias (%)	-6.0	7.8	14.1	10.8	15.7				
	800	CV (%)	6.9	0.3	2.0	2.6	0.7	796.8	-0.4	4.1	4.2
		Bias (%)	-3.3	-3.9	-2.0	1.6	4.6				
Dihydro-*N*-ethylhexedrone	30	CV (%)	2.0	3.6	2.6	0.6	3.8	29.5	-1.6	5.1	5.3
		Bias (%)	5.0	1.4	-2.3	-1.3	-8.6				
	400	CV (%)	1.4	3.7	1.4	1.2	2.1	435.2	8.8	4.7	4.9
		Bias (%)	-1.8	7.8	10.6	11.5	12.3				
	800	CV (%)	3.5	1.9	2.6	4.1	4.0	801.9	0.2	5.1	5.2
		Bias (%)	-1.4	-7.0	2.9	3.0	3.1				
Dihydro-dibutylone	30	CV (%)	3.3	3.9	7.7	8.9	4.6	29.4	-1.9	8.2	8.3
		Bias (%)	-2.1	-6.2	9.1	-5.3	-4.9				
	400	CV (%)	3.6	2.2	1.0	2.0	1.6	427.4	6.8	7.9	8.2
		Bias (%)	-8.5	12.7	13.7	11.0	0.3				
	800	CV (%)	1.0	1.2	0.2	1.0	1.7	800.1	0.0	2.9	3.0
		Bias (%)	1.3	-4.1	-1.7	2.7	2.3				
Dihydro-*N*-ethylpentylone	30	CV (%)	8.7	7.8	2.5	7.9	2.2	29.1	-2.9	8.3	8.4
		Bias (%)	7.4	-9.3	2.0	-2.1	-8.9				

	400	CV (%)<br>Bias (%)	4.6<br>-4.9	0.4<br>5.3	3.2<br>7.7	7.0<br>12.8	2.9<br>13.0	430.5	7.6	6.7	6.9
	800	CV (%)<br>Bias (%)	0.3<br>-11.9	1.5<br>-0.8	4.6<br>-4.2	2.6<br>7.5	2.3<br>2.1	794.3	-0.7	6.7	7.0
Dihydro-4-MPD	30	CV (%)<br>Bias (%)	2.8<br>18.3	4.5<br>3.0	6.0<br>3.6	5.0<br>-1.4	2.7<br>6.6	31.5	5.2	7.0	7.1
	400	CV (%)<br>Bias (%)	1.4<br>-9.4	8.1<br>-1.7	1.6<br>3.5	7.2<br>5.3	9.6<br>15.7	414.2	3.5	9.9	10.1
	800	CV (%)<br>Bias (%)	0.1<br>-1.4	1.4<br>-8.3	3.6<br>2.8	3.9<br>0.7	5.7<br>6.4	801.1	0.1	6.1	6.3
Dihydro-4-CEC	30	CV (%)<br>Bias (%)	6.8<br>15.3	6.8<br>0.3	9.4<br>7.6	3.0<br>-3.0	5.2<br>0.0	31.0	3.2	8.3	8.4
	400	CV (%)<br>Bias (%)	1.7<br>6.0	2.1<br>12.9	3.5<br>13.0	1.7<br>12.7	4.0<br>-4.4	432.8	8.2	7.1	7.3
	800	CV (%)<br>Bias (%)	0.0<br>1.8	1.3<br>-2.3	1.3<br>-2.2	2.0<br>1.4	1.5<br>-0.7	795.6	-0.5	2.2	2.2
Dihydro-4-F-PHP	30	CV (%)<br>Bias (%)	10.2<br>-3.0	6.9<br>-17.8	1.2<br>-15.7	0.2<br>-12.0	1.0<br>-17.2	25.8	7.2	7.2	7.4
	400	CV (%)<br>Bias (%)	11.6<br>-11.6	2.4<br>4.1	15.0<br>-1.9	8.3<br>6.4	0.9<br>10.9	410.1	2.5	10.2	10.4
	800	CV (%)<br>Bias (%)	0.4<br>0.0	5.7<br>-5.1	4.8<br>0.5	4.0<br>0.5	4.7<br>4.8	801.1	0.1	5.1	5.1

Table S3. Group A and B of controls comprising of common drugs (n=196) at a concentration of 10 or 100 ng/mL (depending on the drug).

Analytes in Group A			
• Acebutolol	• Tibolone	• Cathine	• Modafinil
• Alprenolol	• Zilpaterol	• Ephedrine	• Nikethamide
• Atenolol	• Bambuterol	• Pholedrine	• Norfenfluramine
• Betaxolol	• Fenoterol	• Cropropamine	• Pemoline
• Bisoprolol	• Formoterol	• Crotethamide	• Cyclazodone
• Bopindolol	• Salbutamol	• Etamivan	• Famprofazone
• Bunolol	• Terbutaline	• Etilefrine	• Pentetrazol
• Carteolol	• Aminoglutethimide	• Fencamfamine	• Phendimetrazine
• Carvedilol	• Anastrazole	• Fencamine	• Prolintane
• Celiprolol	• Testolactone	• Fenetylline	• Ritalinic acid
• Esmolol	• Tamoxifen	• Fenfluramine	• Selegeline
• Labetalol	• Toremifene	• Fenproporex	• Sibutramine
• Metipranolol	• Amiloride	• Heptaminol	• Strychnine

• Metoprolol	• Bumetanide	• Hydroxybromant.	• Dimethylamphetamine
• Nadoxolol	• Canrenone	• Isometheptene	• Mephentermine
• Oxprenolol	• Chlorexolone	• Methylenedioxyamphetamine	• Dobutamine
• Pindolol	• Clopamide	• Ecstasy	• Buprenorphine
• Propranolol	• Indapamide	• Mefenorex	• Dextromoramide
• Sotalol	• Triamterene	• Mesocrab	• Fentanyl
• Timolol	• Amfepramone	• Methamphetamine	• Hydromorphone
• Stanozolol	• Amiphenazole	• Ortetamine	• Hydrocodone
• Danazol	• Amphetamine	• Phentermine	• Morphine
• Gestrinone	• Benzoylecgonine	• Methoxyphenamine	• Codeine
• Metribolone	• Benzphetamine	• Methylephedrine	• Methadone
• Tetrohydrogestrinone	• Benzylpiperazine	• Methylhexaneamine	• Oxycodone
• Clenbuterol	• p-OH amphetamine		• Pentazocine
Continued			

• Oxymorphone	• Prednisone	• FPCAM	• Chlorthalidone
• Pethidine	• Methylprednisolone	• Triamcinolone	• Dichlorphenamide
• Beclomethasone	• Deflazacort	• Bupropion	• Etacrynic acid
• Betamethasone	• Desacetyl deflazacort	• Tramadol	• Furosemide
• Budesonide	• Desonide	• Ketoconazole	• Methylphenidate
• OH-prednisolone	• Fludrocortisone	• Andarine	
• 6b-OH budesonide	• Flumethasone	• Ostarine	
• Clobetasol	• Triamcinolone acetonide	• Acetazolamide	
• Prednisolone	• Fluticasone proprionate	• Chlorothiazide	
Analytes in Group B			
• Efaproxiral	• Fenbutrazate	• Pipradrol	• Cyclothiazide
• Acetylcarnitine	• Phenpromethamine	• Mitragynine	• Epitizide
• Diacetolol	• Para-methylamphetamine	• OH-mitragynine	• Polythiazide
• Nadolol	• Tuaminoheptane	• Fluconazole	• Trichloromethiazide
• OH-propranolol	• Adrafinil frag	• Miconazole	• Altizide

- Exemestane
- Sameterol
- Clomiphene
- Fulvestrant
- Mefruside
- Eplerenone
- Piretanide
- Amphetaminil
- Benfluorex
- Carphedon
- Clobezorex
- Oxilofrine
- Propylhexedrine
- Etilamphetamine
- Ethylphenylbutylamine
- Prenylamine
- Mephedrone
- Trimetazidine
- 1,3-Dimethoxybenzene
- 2-am-6-me-heptane
- 6-Monoacetylmorphine
- Dexamethasone
- Flunisolide
- Fluocortolone
- Itraconazole
- *N,N*-Dimethyltryptamine
- Selective Androgen Receptor Modular- 4F
- Letrazole metab.
- Selective Androgen Receptor Modular 4-Cl
- Probenecid
- Bendroflumethazide
- Metolazone
- Benzthiazide
- Cyclopenthiazide
- Butizide
- Methyclothiazide
- Xipamide
- Torasemide
- Hydroflumethiazide

Table S4. Processed sample stability of SCt in urine after 24, 48 and 72 h of storage on autosampler (10°C) at QC low (30 ng/mL) and QC high (800 ng/mL). (n = 3).

	24 h		48 h		72 h	
	QC low	QC high	QC low	QC high	QC low	QC high
Analyte	% loss (RSD)	% loss (RSD)	% loss (RSD)	% loss (RSD)	% loss (RSD)	% loss (RSD)
Mephedrone	-1.1 (1.0)	1.0 (3.6)	-2.9 (1.8)	0.4 (2.3)	-2.0 (2.7)	-1.1 (1.0)
Methylone	9.2 (5.2)	0.0 (3.4)	-8.3 (7.0)	-5.1 (2.4)	-8.1 (12.6)	9.2 (5.2)
Methedrone	-6.2 (18.3)	-11.6 (2.2)	-8.5 (15.6)	-11.9 (4.9)	-12.3 (14.6)	-6.2 (18.3)
Ethylone	-7.1 (6.2)	1.7 (2.4)	-12.2 (6.6)	-1.7 (4.0)	-14.5 (6.8)	-0.4 (4.5)
Butylone	3.8 (2.6)	-5.9 (8.8)	-14.2 (17.0)	-13.0 (5.4)	-12.7 (8.9)	-11.7 (3.1)
Dibutylone	5.7 (3.5)	0.2 (2.9)	4.1 (6.5)	-0.3 (1.8)	-6.5 (5.2)	-1.3 (1.1)
4-CEC	0.3 (8.1)	-8.5 (8.8)	5.1 (14.3)	-2.5 (9.8)	2.0 (4.3)	-3.5 (4.4)
4-Cl- α -PPP	-2.2 (6.6)	-5.5 (3.1)	3.2 (8.4)	-2.2 (1.2)	1.4 (8.9)	-4.8 (2.2)
N-ethylpentylone	0.1 (9.6)	-9.2 (8.5)	-8.5 (10.6)	-4.3 (2.0)	-2.1 (7.2)	-11.4 (0.6)
4-EMC	-9.4 (14.4)	-6.8 (9.8)	-6.6 (14.9)	2.1 (2.0)	-7.0 (6.3)	-1.2 (7.8)
α -PVP	-3.2 (4.8)	-10.8 (3.3)	9.9 (14.3)	-5.6 (1.5)	0.4 (3.1)	-13.3 (2.1)

MDPV	3.6<br>(8.0)	-8.8<br>(17.3)	5.6<br>(8.9)	0.3<br>(3.7)	1.7<br>(8.0)	-1.2<br>(7.4)
4-MPD	1.5<br>(6.9)	-0.7<br>(5.3)	-3.0<br>(13.9)	-1.0<br>(2.8)	-0.5<br>(5.5)	-10.5<br>(6.6)
N-ethylhexedrone	-3.6<br>(13.1)	-6.5<br>(2.7)	-5.9<br>(12.2)	1.8<br>(1.0)	-5.8<br>(6.1)	-5.7<br>(2.1)
4-F-PHP	-7.7<br>(12.8)	-9.0<br>(4.8)	-6.5<br>(3.4)	-2.4<br>(0.3)	-13.8<br>(3.9)	-11.0<br>(0.6)
4-Cl-α-PVP	-10.1<br>(17.4)	-7.0<br>(14.4)	-12.6<br>(12.0)	-4.8<br>(2.9)	-16.1<br>(7.4)	-11.8<br>(3.0)
Dihydro-mephedrone	-12.5<br>(11.5)	-5.4<br>(5.0)	-17.3<br>(15.0)	-4.2<br>(5.0)	-15.1<br>(12.5)	-2.4<br>(1.9)
Dihydro-MDPV	-13.0<br>(15.0)	-10.3<br>(8.3)	-7.7<br>(11.9)	1.3<br>(5.3)	-18.0<br>(6.3)	-5.4<br>(8.5)
Dihydro-4-Cl-α-PPP	8.8<br>(15.6)	-3.4<br>(2.3)	10.8<br>(10.3)	-4.0<br>(4.3)	6.8<br>(14.8)	-6.6<br>(3.1)
Dihydro-4-EMC	-1.9<br>(3.3)	-5.7<br>(2.7)	-0.7<br>(17.6)	3.2<br>(1.7)	-17.0<br>(6.3)	-3.9<br>(2.3)
Dihydro-*N*-ethylhexedrone	-3.9<br>(11.1)	-3.8<br>(6.1)	2.0<br>(16.5)	6.6<br>(3.0)	-1.6<br>(3.3)	-2.1<br>(0.9)
Dihydro-dibutylone	-3.8<br>(11.8)	-7.9<br>(6.8)	4.8<br>(13.9)	-6.5<br>(2.0)	-8.6<br>(21.0)	-8.0<br>(1.8)
Dihydro-*N*-ethylpentylone	-3.0<br>(2.1)	-9.1<br>(3.9)	-5.4<br>(6.2)	-5.6<br>(3.0)	-1.7<br>(5.9)	-9.4<br>(2.5)
Dihydro-4-MPD	3.9<br>(13.8)	-5.3<br>(10.9)	0.7<br>(15.1)	-2.2<br>(2.9)	1.6<br>(8.5)	-7.9<br>(1.1)
Dihydro-4-CEC	-3.2<br>(11.1)	-4.7<br>(5.3)	5.5<br>(4.0)	-1.8<br>(2.8)	-3.0<br>(6.1)	-4.1<br>(2.0)
Dihydro-4-F-PHP	-8.8<br>(6.3)	-12.7<br>(13.0)	-3.3<br>(11.8)	-9.2<br>(7.7)	-5.4<br>(12.5)	-10.9<br>(1.9)

Table S5. Calculated ion ratio tolerance between less intense peak area to that of more intense and that was established by the spiked QC samples for metabolites positive findings.

	Ion ratio masses	Calculated ion ratio	Expected ion ratio	Ion ratio tolerance	Conforms
Dihydro-mephedrone (Sample No. 19)	147/162	0.08	0.11	– 27.3%	Yes
Dihydro-*N*-ethylpentylone (sample No. 34)	191/234	0.19	0.15	26.7%	Yes
Dihydro-mephedrone (sample No. 49)	147/162	0.13	0.11	18.2%	Yes

Table S6. Maximum permitted tolerance for ion ratio.

**Ion ratio**	**Permitted tolerance**
> 0.50	± 20%
0.20 – 0.50	± 25%

0.10 – 0.20	± 30%
< 0.10	± 50%

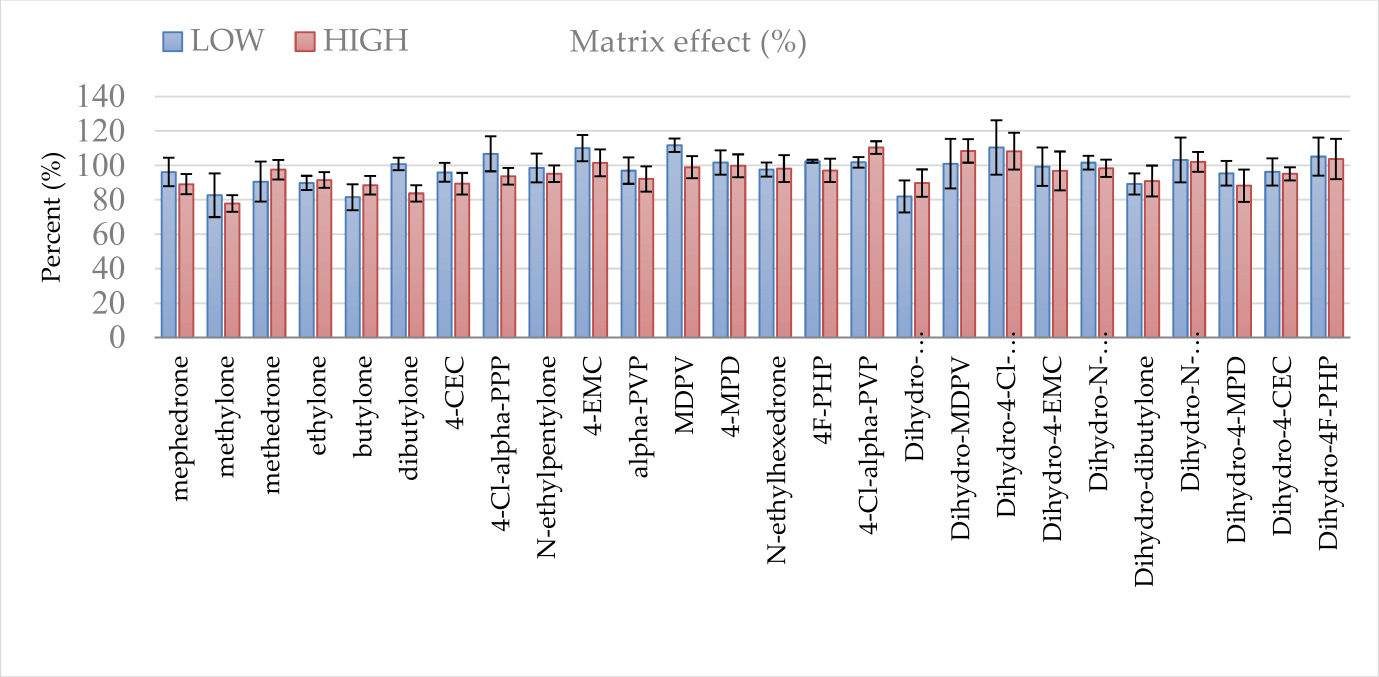

Figure S1. Matrix effects of SCt and metabolites in urine at 30 and 800 ng/mL. The error bars show RSD (n=5).

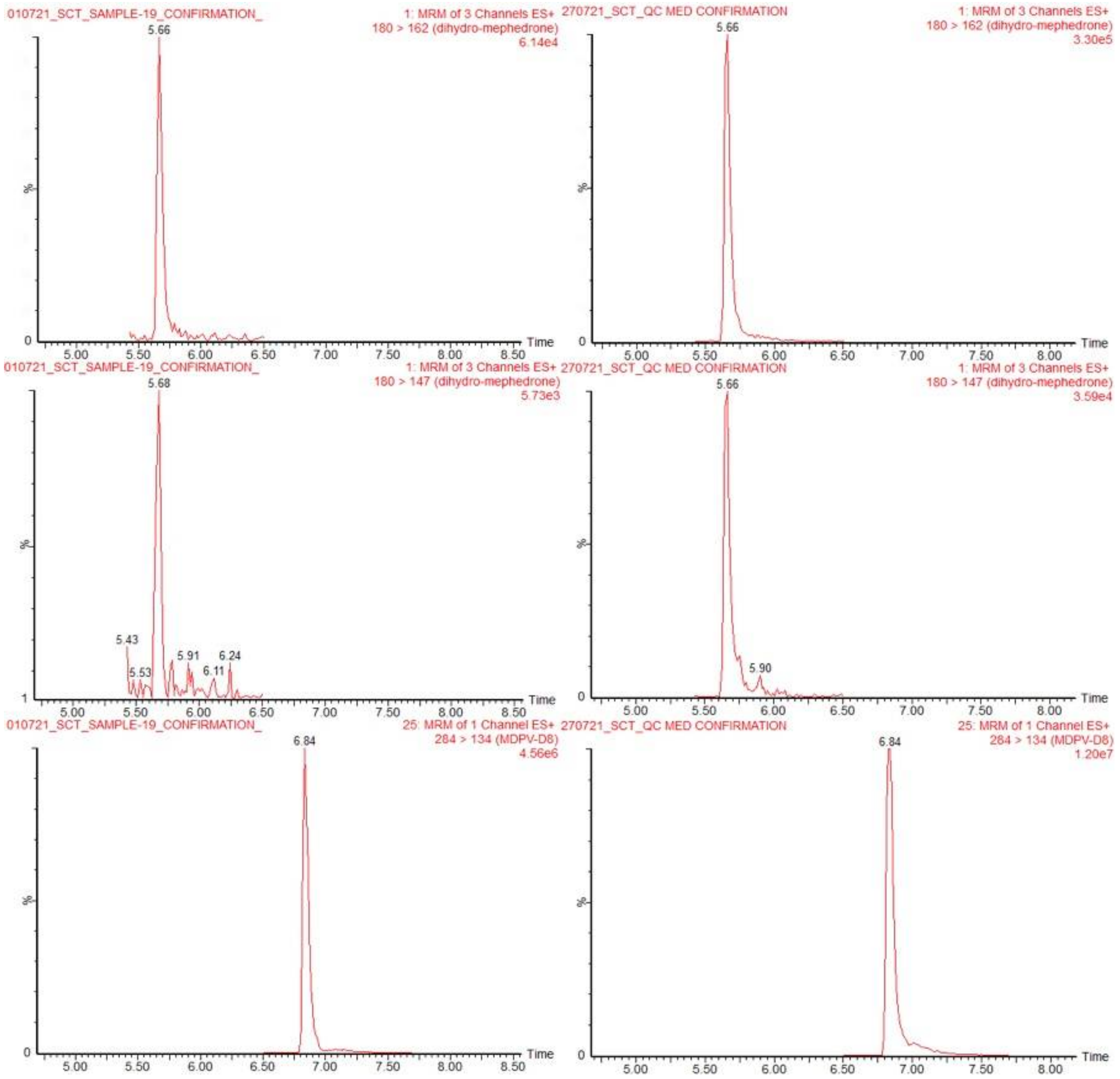

Figure S2. Confirmatory results for an authentic urine sample (No. 19) testing positive for dihydro-mephedrone (left); and a QC sample (400 ng/mL; right).

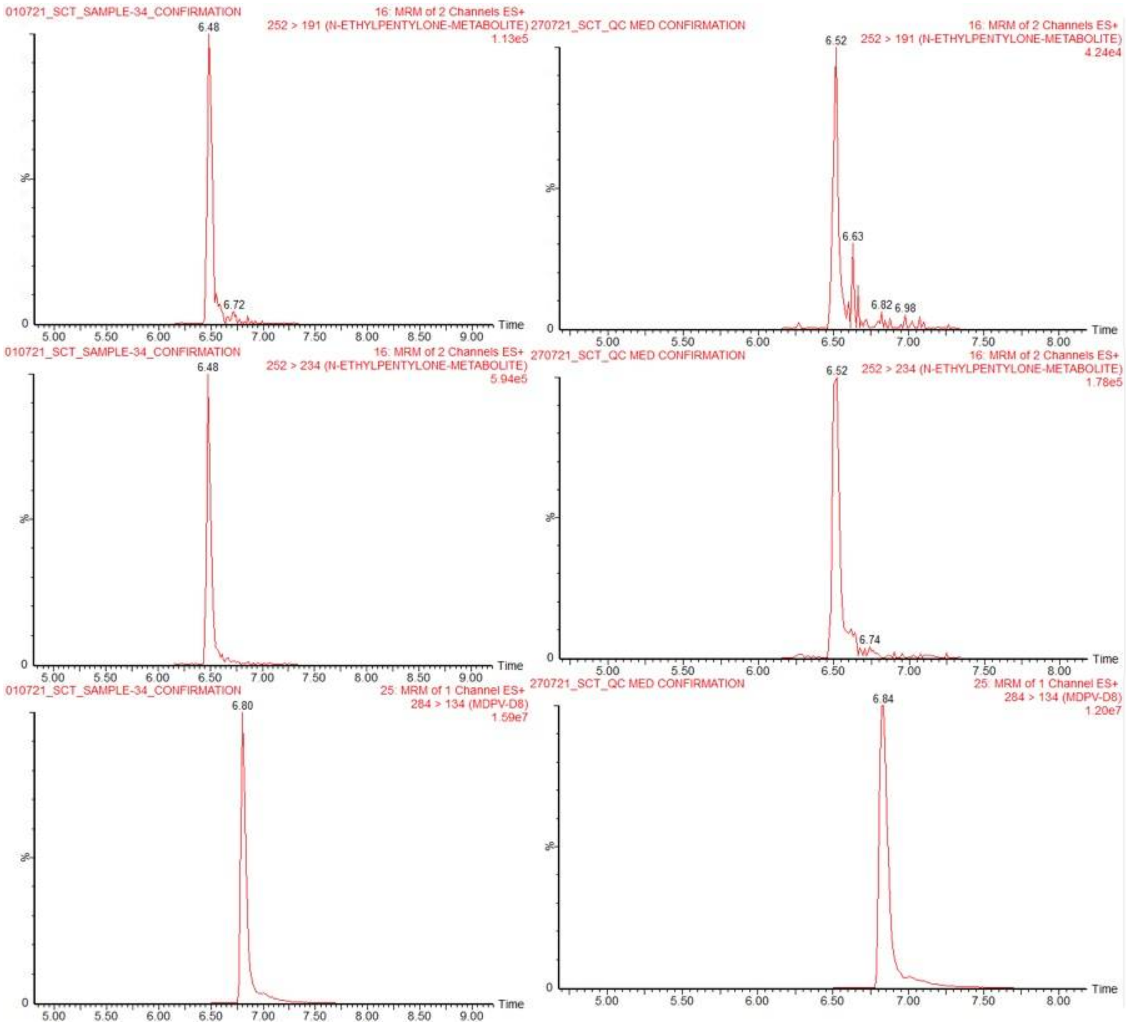

Figure S3. Confirmatory results for an authentic urine sample (No. 34) testing positive for dihydro-*N*-ethylpentylone (left); and a QC sample (400 ng/mL; right).

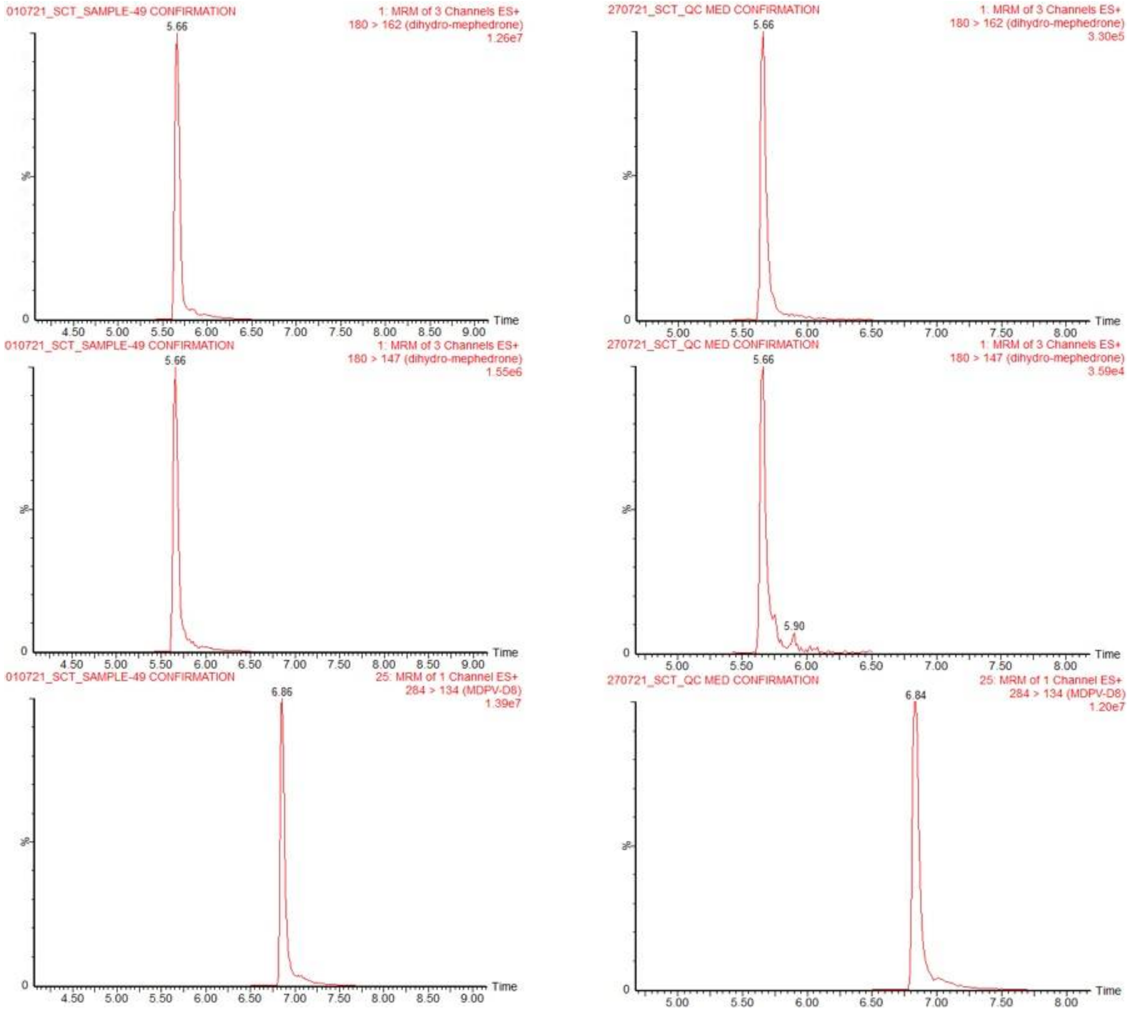

Figure S4. Confirmatory results for an authentic urine sample (No. 49) testing positive for dihydro-mephedrone (left); and a QC sample (400 ng/mL; right).

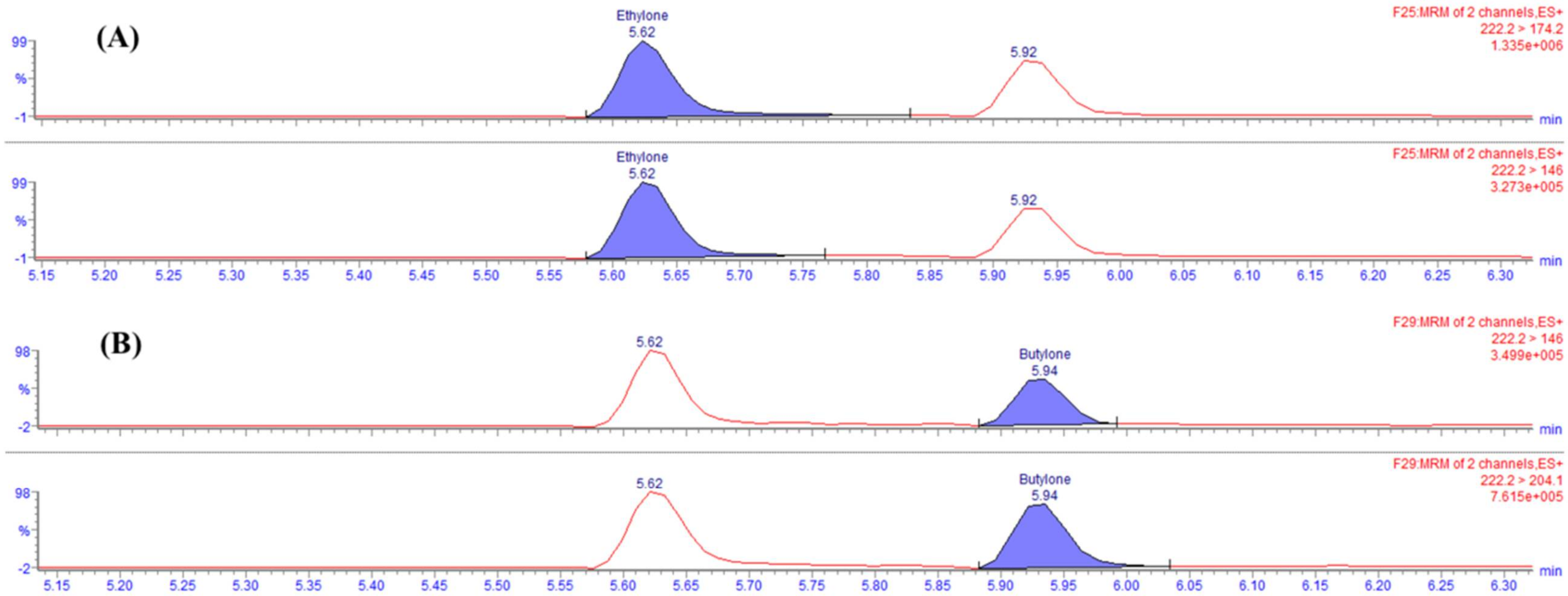

Figure S5. Representative MRM chromatograms of ethylone (A) and butylone (B).

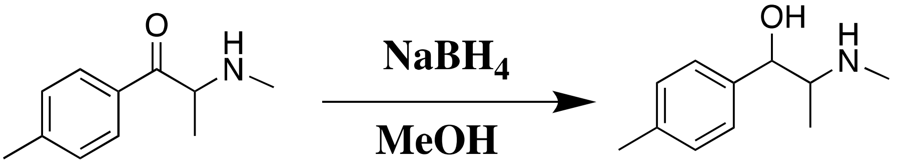

Mephedrone **Dihydro-mephedrone (metabolite)**

Figure S6. Reduction of mephedrone to dihydro-mephedrone.
